# Pattern blending enriches the diversity of animal colorations

**DOI:** 10.1126/sciadv.abb9107

**Published:** 2020-12-02

**Authors:** Seita Miyazawa

**Affiliations:** Graduate School of Frontier Biosciences, Osaka University, Osaka, Japan. Email: seita@fbs.osaka-u.ac.jp

## Abstract

Animals exhibit a fascinating variety of skin patterns, but mechanisms underlying this diversity remain largely unknown, particularly for complex and camouflaged colorations. A mathematical model predicts that intricate color patterns can be formed by “pattern blending” between simple motifs via hybridization. Here, I analyzed the skin patterns of 18,114 fish species and found strong mechanistic associations between camouflaged labyrinthine patterns and simple spot motifs, showing remarkable consistency with the pattern blending hypothesis. Genomic analyses confirmed that the coloring on multiple labyrinthine fish species has originated from pattern blending by hybridization, and phylogenetic comparative analyses have further substantiated the pattern blending hypothesis in multiple major fish lineages. These findings provide a plausible mechanistic explanation for the characteristic diversity of animal markings and suggest a novel evolutionary process of complex and camouflaged colorations by means of pattern blending.

## Teaser

Comprehensive analyses of fish colorations illuminate a simple mechanism creating intricate patterns on animals.

## INTRODUCTION

**Fig. 1:**
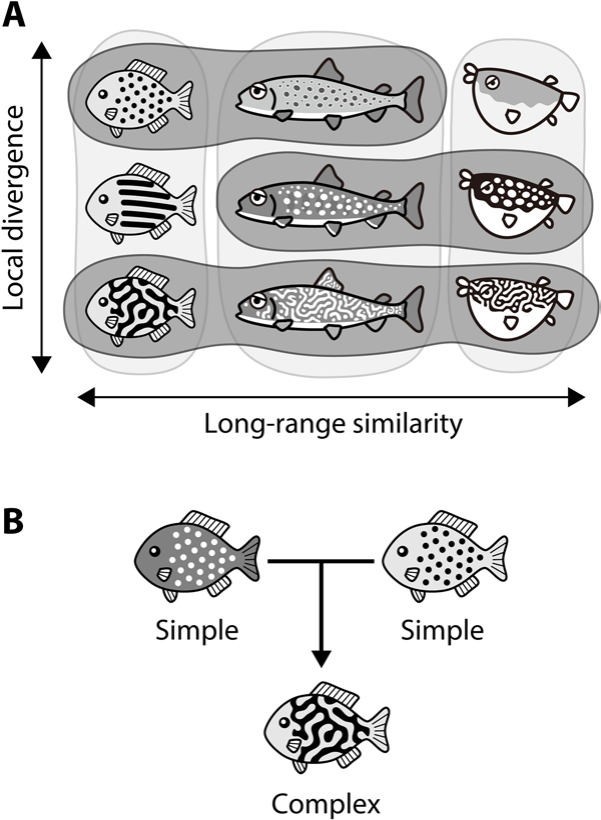
Characteristic diversity of animal color patterns and “pattern blending” hypothesis. (**A**) Animal colorations are often more similar among distantly related species (horizontal) than among closely related species (vertical), showing a characteristic diversity―long-range similarity over local divergence. (**B**) Pattern blending hypothesis. Mathematical models predict that complex patterns can be formed by pattern blending between simple motifs via hybridization.

Animal color patterns play critical roles in animal survival, adaptation, intra- and interspecific communication, and speciation (*1*–*6*). Traditionally, and reasonably, biologists have used animal coloration as an important key to identification of a species (*7*, *8*). We often see apparent discreteness or qualitative differences in color patterns even between closely related animals (*9*–*13*) and recognize these differences as strong evidence for novel/distinct species. At the same time, many color pattern motifs―from simple spot or stripe patterns to complex and camouflaged labyrinthine patterns―appear repeatedly across a wide range of taxonomic groups (*14*–*16*). As a result, we see many cases in which a certain animal can more closely resemble distantly related species than its own close relatives (Fig. 1A). This extraordinary level of homoplasy―ubiquitous, long-range similarity over local divergence―is characteristic of animal coloration, which is rarely seen in other morphological traits, such as size and shape of body parts.

The evolutionary and developmental mechanisms underlying this peculiar, cross-taxonomic diversity of animal color patterns have remained largely unexplored until recently. In the past few years, however, progress has been made by identifying genes that are involved in similar skin patterns across multiple animal species (*14*–*16*). Some simple motifs, such as longitudinal stripes and wing spots, have been shown to be formed by using genetically encoded prepatterns originating from spatial landmarks or structural components of other body parts (*12*, *17*). Accordingly, with respect to these simple pattern motifs, intuitive explanations for their repeated occurrence across clades have been provided: Within each lineage, the painting of prepatterned regions is just switched on and off by particular genes (*12*, *15*). By contrast, more complex colorations such as camouflaged labyrinthine patterns have apparently no obvious relationship with any structures or body parts and cannot be formed simply from prepatterned positional information. Hence, it still remains a mystery how these intricate patterns have evolved and why unexpectedly similar, elaborate pattern motifs repeatedly and ubiquitously emerged across distantly related animal taxa.

A theoretical approach (*18*) has suggested an intriguing possibility to unravel this mystery. Using a specific class of the reaction-diffusion (RD) system (*19*) first proposed by Alan Turing (*20*), it has been predicted that complex and camouflaged markings can be formed by the morphological “blending” of simple patterns by hybridization (*18*). For example, crossing between virtual organisms with inverted spot patterns―one with light spots on a dark background and the other with dark spots on a light background―will necessarily result in intricate labyrinthine patterns as morphologically “blended” intermediate phenotypes (Fig. 1B and fig. S1).

If this prediction is widely applicable to real animals, one can expect that organisms having complex and camouflaged motifs can abruptly emerge from events of hybridization between species that have simple patterns. This “pattern blending” hypothesis would account for the enigmatic repeated occurrence of similar, intricate pattern motifs across distantly related taxa, while simultaneously offering a plausible explanation for the discrete, qualitative pattern differences between closely related species. Although the model prediction has been partially confirmed by empirical evidence such as artificial hybridization of salmonid species (*18*), natural instances supporting the pattern blending hypothesis so far are very limited, and it is unclear whether, or to what extent, pattern blending contributes to the actual diversity of animal color patterns.

In this study, I test the pattern blending hypothesis by exploring the natural diversity of color patterns in fish lineages. First, I analyze the skin patterns of more than 18,000 species of marine and freshwater fishes and examine phylogenetic collocations of pattern motifs to infer the mechanistic associations among them. I show that apparent phenotypic similarity does not necessarily correspond to the mechanistic association between pattern motifs. On the contrary, strong positive associations between phenotypically dissimilar pattern motifs are present, which are consistent with the pattern blending hypothesis. Next, I provide demonstrative examples of pattern blending by using color pattern quantification and comparative genomic analyses targeting representative fish species. Last, by applying phylogenetic comparative methods, I show that pattern blending has contributed to the diversity of color patterns in multiple major fish lineages.

## RESULTS

### Diversity of color patterns in marine and freshwater fish

**Fig. 2:**
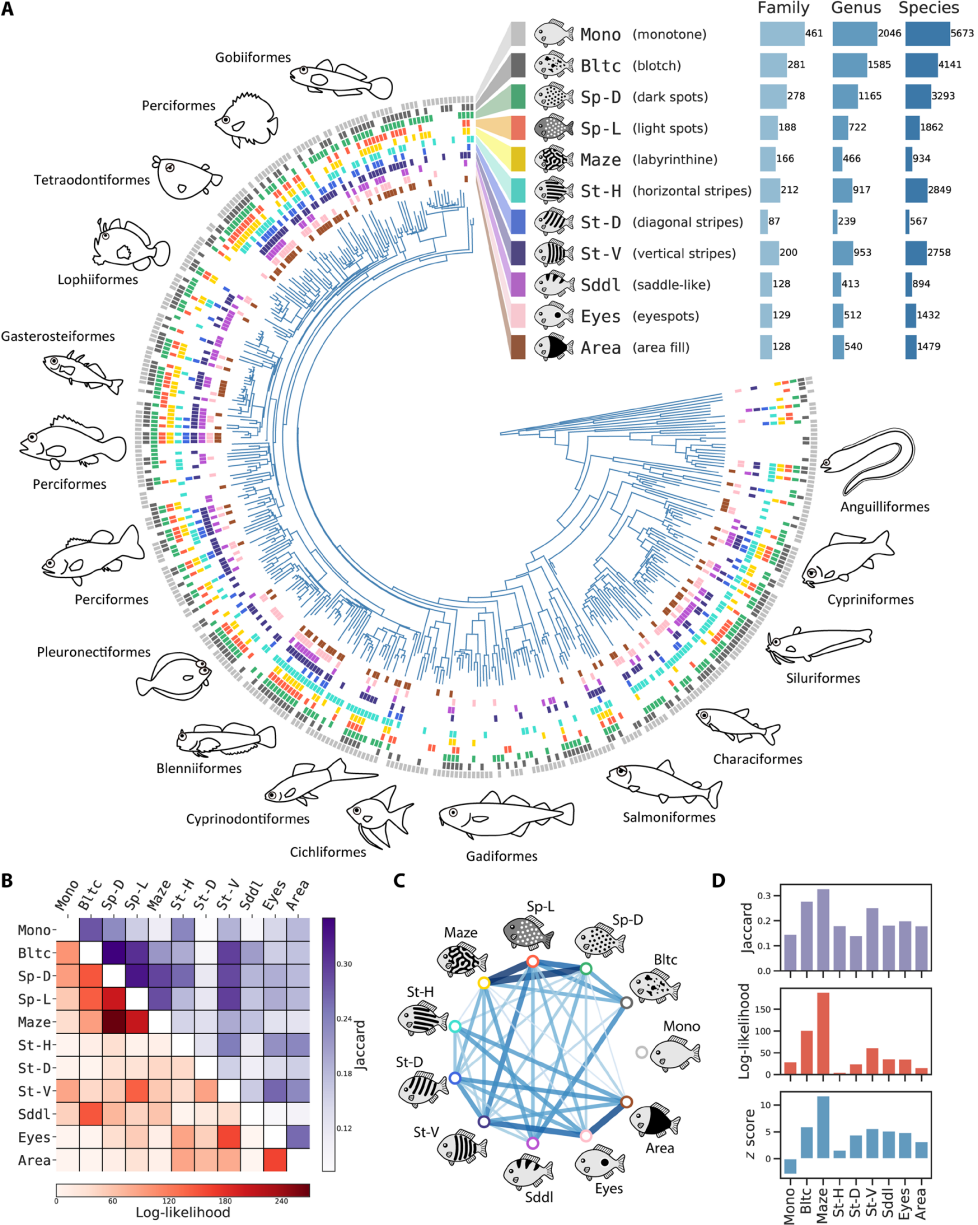
Fish color pattern diversity and mechanistic associations among pattern motifs. (**A**) Pattern diversity in marine and freshwater fish families. The occurrence of the pattern motifs in each fish family is indicated by colored bars at the tip of the tree. Each pattern motif is widespread across fish lineages, showing the ubiquitous long-range similarity over local divergence. The 11 classes of pattern motifs are illustrated along with their abbreviations, color codes, and numbers of occurrence (inset). A total of 559 families, 4069 genera, and 18,114 species were examined. Fish phylogeny was based on Betancur-R *et al.* (*61*). (**B**) Heatmap showing the strength of the genus-level co-occurrence between each pairwise combination of the 11 pattern motifs. (**C**) Mechanistic associations among pattern motifs evaluated using *z* score. (**D**) Triple co-occurrence analysis showing the strength of the phylogenetic collocation of each pattern motif with a set of two spot motifs (Sp-D and Sp-L).

To examine the diversity of color patterns in fish lineages, I performed pattern annotation on images of 18,114 fish species referenced from public databases (FishBase: https://www.fishbase.org/ and FishPix: http://fishpix.kahaku.go.jp/fishimage-e/). I used 11 well-recognized classes of pattern motifs for annotation (*7*, *8*): 4 types of stripe patterns (St-H, horizontal stripes; St-D, diagonal stripes/bands; St-V, vertical stripes/bars; Maze, labyrinthine stripes), 3 types of spot patterns (Sp-D, small dark spots on a light background; Sp-L, small light spots on a dark background; Eyes, eyespots/spots larger than eyes), and 4 other patterns (Area, area fill pattern; Sddl, saddle-like pattern; Bltc, blotch/speckle pattern; Mono, monotone/patternless). Each image was binary labeled for each class of pattern motifs using a web-based in-house annotation system, and the presence/absence of each pattern motif for each species and taxonomic group was recorded (Fig. 2A, fig. S2, and data file S1). The compiled data revealed that all of the 11 color pattern motifs were widespread across the lineages, illustrating the ubiquitous long-range similarity over local divergence, although the range and abundance varied among the pattern motifs (Fig. 2A).

Using this dataset, I examined the mechanistic associations among color pattern motifs by evaluating their phylogenetic co-occurrences. It is reasonable to assume that mechanistically related motifs, wherein pattern transition can readily occur, are likely to co-occur within each small taxonomic group, such as genus. Then, the mechanistic association between pattern motifs can be inferred based on this “phylogenetic collocation” (i.e., within-genus co-occurrence of pattern motifs). This association should not necessarily or simply be a measure of superficial, phenotypic similarity between pattern motifs; rather, it should reflect the underlying mechanistic connections related to evolutionary, developmental, or ecological constraints and/or structures. I used three different association measures to evaluate the within-genus co-occurrence between each pairwise combination of the 11 classes of color pattern motifs (Fig. 2, B and C). The Jaccard index (JI) has been classically used for evaluating similarities between two sets of binary data in many fields, including community ecology (*21*). The log-likelihood (LL) and *z* score (*Z*) have been frequently used for quantifying collocational strength in the field of corpus linguistics (*22*). According to these indices, strong associations were present among spot-like motifs (Sp-D, Sp-L, and Bltc; JI = 0.31 to 0.40, LL = 144 to 203, *Z* = 6.9 to 9.7), which are concordant with their phenotypic similarity. In contrast, despite the obvious morphological similarity among stripe motifs (St-H, St-D, St-V, and Maze), their associations were relatively weak, if any (JI = 0.13 to 0.24, LL = 9.8 to 88, *Z* = 2.4 to 7.7). This implies that developmental and/or ecological constraints may exist within each class of stripe motifs, so that the transition to another stripe class is restricted. For example, the development of horizontal stripes in fish may depend heavily on prepatterns related to structural body parts (*9*), as is the case in mammals and birds (*14*, *17*).

The most notable associations were found in distinctly different motif pairs: labyrinthine and spot motifs (Maze and Sp-D: JI = 0.29, LL = 269, *Z* = 11.8; Maze and Sp-L: JI = 0.28, LL = 207, *Z* = 12.1) (Fig. 2, B and C). Considering the apparent phenotypic differences between labyrinths and spots, their strong association seems counterintuitive in terms of morphology. On the other hand, it is consistent with the pattern blending hypothesis and in silico hybridization experiments (Fig. 1B and fig. S1). This consistency is more evident in triple co-occurrence analyses of pattern motifs: When focusing on the genera in which dark (Sp-D) and light (Sp-L) spots co-occur, the pattern motif most strongly and substantially associated with these two spot motifs was indicated to be the labyrinthine motif (Maze) by all three association measures (Fig. 2D). To confirm these results, I used three other measures [Sørensen-Dice coefficient (SDC), Simpson similarity index (SSI), and *T* score (*T*)] and obtained qualitatively similar results (fig. S3). By contrast, for motif pairs other than dark and light spots, the strongest association was detected with motifs other than the labyrinthine patterns (fig. S4).

### Pattern quantification and genomic analyses of pufferfish

**Fig. 3:**
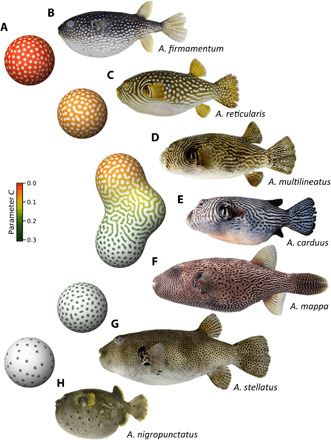
Pattern variations in model simulation and actual animals. (**A**) Patterns generated by computer simulation based on an RD system. Spheres represent “pure species” computed with uniform parameter values. A fused blob represents a “hybrid” resulting from in silico hybridization. Color hue and lightness indicate the parameter value and the concentration of the core factor in the model, respectively. (**B** to **H**) Body patterns of *Arothron* species: (B) *A. firmamentum* (KPM-NI 28845), (C) *A. reticularis* (KPM-NI 31898), (D) *A. multilineatus* (KAUM-I. 13606), (E) *A. carduus* (HUMZ 35438), (F) *A. mappa* (NIFREL), (G) *A. stellatus* (KAUM-I. 52638), and (H) *A. nigropunctatus* (KAUM-I. 39888). Photographs by H. Senou, Kanagawa Prefectural Museum of Natural History (B and C), The Kagoshima University Museum (D, G, and H), K. Matsuura (E), and S. Miyazawa (F).

**Fig. 4:**
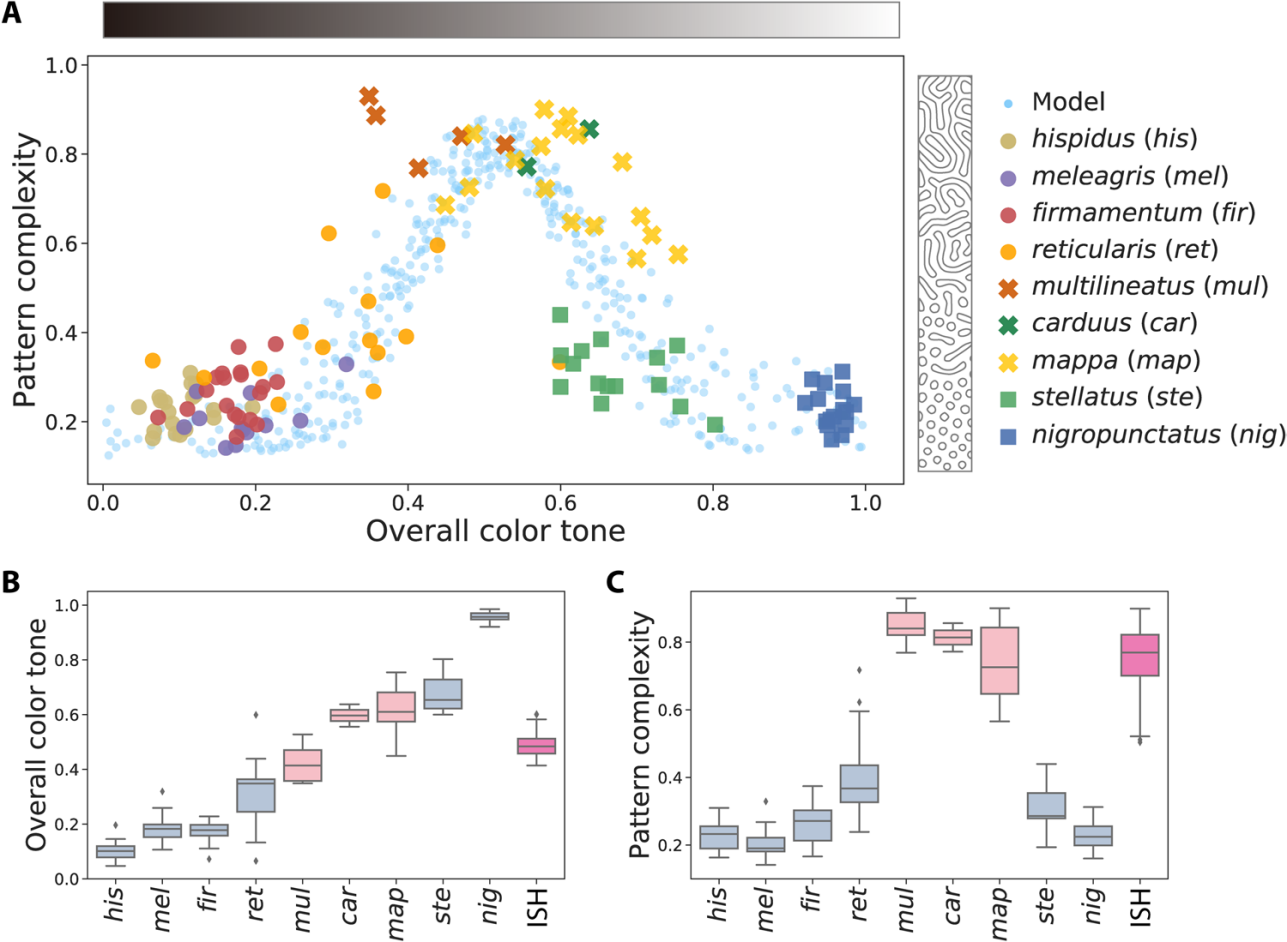
Quantification of color patterns of simulated and real animals. (**A**) The complexity of each color pattern (measured using element-wise circularity; see Materials and Methods) plotted against the overall color tone of the pattern (pattern lightness measured as the proportion of unpigmented area in a binarized image). Circles, squares, and crosses indicate light spotted, dark spotted, and labyrinthine species, respectively. Blue dots denote simulated patterns using the RD model. (**B** and **C**) Box plots showing overall color tone (B) and pattern complexity (C) of *Arothron* species and in silico hybrids (ISH). Light blue, pink, and magenta colors indicate spotted species, labyrinthine species, and ISH, respectively. The box extends from the lower to upper quartile values of the data, with a line at the median. The whiskers and points are 1.5× interquartile range and outliers, respectively.

To investigate the possibility of pattern blending in more detail, I performed an in-depth analysis of the skin pattern diversity in a representative small taxonomic group: pufferfish species of the genus *Arothron*, which are known for the compact size of their genome (less than 400 Mb, smallest among vertebrates), as well as their remarkable and diverse color patterns (Fig. 3) (*23*, *24*). Pattern quantification targeting a total of 120 individuals from nine species revealed that pattern variations observed among *Arothron* fishes were in good agreement with numerical simulations based on the RD model (Fig. 4A, figs. S5 and S6, and data file S1). Above all, the labyrinthine patterns of *Arothron* species, *Arothron multilineatus* Matsuura, 2016, *Arothron carduus* (Cantor, 1849), and *Arothron mappa* (Lesson, 1831) (Fig. 3, D to F) were shown to have “intermediate” color tones between two types of spot patterns (a darker background tone for light spot patterns and a lighter background tone for dark spot patterns) (Fig. 4B) and a “transgressive” property as to the pattern complexity (labyrinthine patterns being more complex than both of spot patterns) (Fig. 4C).

This twofold character of the labyrinthine patterns of *Arothron* species shows a remarkable correspondence with that of the blended patterns emerging in the in silico hybrids between light spots and dark spots (Fig. 3A and fig. S1). According to the pattern blending hypothesis, these observations lead to a testable prediction: the possible hybrid origin of the labyrinthine *Arothron* species and their putative parental species, one that has light spots on a dark background [e.g., *Arothron firmamentum* (Temminck and Schlegel, 1850) and *Arothron reticularis* (Bloch and Schneider, 1801); Fig. 3, B and C] and the other with dark spots on a light background [e.g., *Arothron stellatus* (Anonymous, 1798) and *Arothron nigropunctatus* (Bloch and Schneider, 1801); Fig. 3, G and H].

To test this prediction, I performed comparative genomic analyses of *Arothron* species. For 12 species of the genus *Arothron*, including 3 labyrinthine species, whole-genome libraries were constructed and sequenced using freshly collected samples and/or museum specimens. I generated a de novo genome assembly of one *Arothron* species, *A. firmamentum* (aroFir_1.0: the draft genome size is 335 Mb, N50 contig and scaffold sizes are 15.9 and 135 kb, respectively), and mapped the reads of 20 individuals from 12 *Arothron* species to this reference genome (mapping rates of all samples ranged from 96 to 98%, except for museum samples). Twenty-eight million single-nucleotide polymorphisms (SNPs) were identified across the *Arothron* species in the form of genotype likelihoods, and complete or nearly complete mitochondrial DNA (mtDNA) genome sequences were also reconstructed from the reads for each species.

**Fig. 5:**
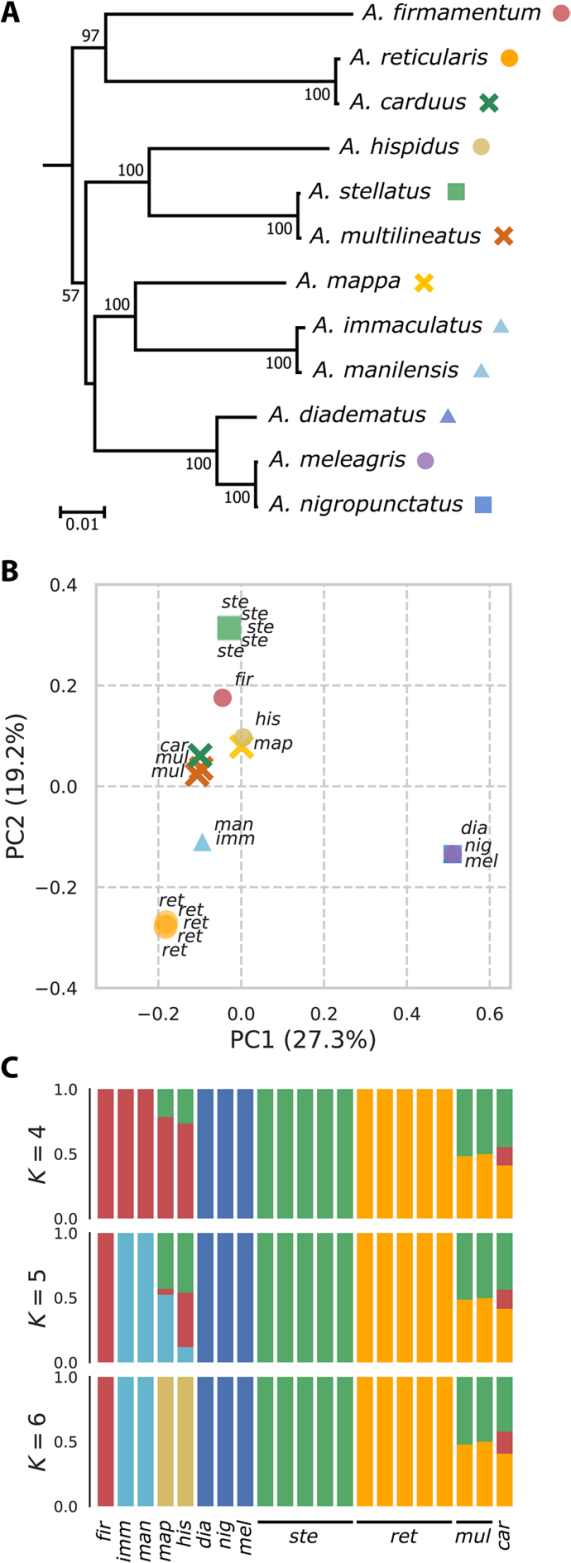
Genomic analyses of *Arothron* species. (**A**) Phylogenetic tree of *Arothron* species based on complete mitochondrial genomes. The numbers indicate the bootstrap percentage. Circles, squares, crosses, and triangles indicate species that have light spot, dark spot, labyrinthine, and other motifs, respectively. (**B**) Principal components analysis based on whole-genome SNP data of 20 individuals from 12 *Arothron* species. (**C**) Admixture plots showing cluster assignments for *K* = 4 to 6 ancestral populations.

According to the phylogenetic tree based on mtDNA genomes (Fig. 5A), the labyrinthine species, *A. carduus*, is very closely related to the white-spotted species, *A. reticularis*, while another labyrinthine species, *A. multilineatus*, appears almost identical to the black-spotted species, *A. stellatus*. On the other hand, whole-genome analyses indicated that these labyrinthine species have mixed ancestry: Principal components analysis (PCA) based on whole-genome SNPs showed that they were both located near the midpoint of the clusters of the white-spotted *A. reticularis* and the black-spotted *A. stellatus* in the plot of the first two components (Fig. 5B). Furthermore, from the admixture analysis based on the same SNP dataset, the ancestry proportion of *A. multilineatus* was estimated to be approximately half of both *A. stellatus* and *A. reticularis* (Fig. 5C). *A. carduus* also showed mixed ancestry between *A. stellatus* and *A. reticularis*, with a small contribution from *A. firmamentum* (Fig. 5C). I also estimated interclass heterozygosity (*25*) based on 5 million diagnostic sites that segregate *A. stellatus* and *A. reticularis*. Most of the callable diagnostic sites were heterozygous in the labyrinthine individuals (*A. multilineatus*: 97 and 99%, *A. carduus*: 87%), indicating that these species are in an early generation of admixture.

These data strongly support the prediction from the pattern blending hypothesis: Two labyrinthine *Arothron* species, *A. multilineatus* and *A. carduus*, are both derived from the interspecific hybridization of the white-spotted *A. reticularis* and the black-spotted *A. stellatus*, although the parental combinations and color patterns were inverted (Fig. 3, D and E; white lines on a black background and black lines on a white background, respectively). According to the original description (*24*, *26*), no morphological traits that can distinguish these two species from other *Arothron* species have been recognized besides the peculiar skin patterns. Their reticulated colorations may, thus, indicate their reticulated origins, rather than their species identities.

As for the other labyrinthine species, *A. mappa*, it seems unlikely that it was in the early generations of admixture (Fig. 5, A to C). However, the ABBA-BABA test (*27*) [Patterson’s *D* statistic and its derivative, *D*_min_ (*28*)] detected traces of past hybridization or introgression between multiple combinations of this and other *Arothron* species, including black-spotted and white-spotted relatives [78% of all possible trios (28 of 36) have a significantly positive *D*_min_ score (Holm-Bonferroni familywise error rate, <0.01); table S1], suggesting the probable occurrence of pattern blending events during the evolution of this *Arothron* species.

### Phylogenetic comparative analysis of color pattern motifs in fish lineages

If pattern blending events have contributed substantially to the evolution and diversity of animal skin patterns, their traces could be detected as signals of correlated evolution between distinct pattern motifs. For example, under the condition of frequent pattern blending, a labyrinthine motif is more likely to emerge in taxonomic groups that contain both dark and light spotted species than in groups without one or both of these spot motifs.

**Fig. 6:**
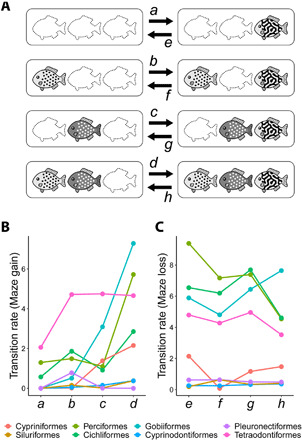
Evolutionary transitions of pattern motifs in major fish orders. (**A**) Schematic example of the pattern motif transitions. The genus-level portfolio of pattern motifs is depicted in rounded rectangles (the presence/absence of the three motifs is shown here). Eight possible transitions between “without Maze” (left) and “with Maze” (right) under the dependent model are indicated by arrows. (**B** and **C**) Transition rates estimated using Bayesian reversible-jump Markov chain Monte Carlo (rjMCMC) approach implemented in BayesTraits (*30*). (B) Maze gain rate. The rates of transition from “without Maze” to “with Maze” [arrows *a* to *d* in (A)]. (C) Maze loss rate [arrows *e* to *h* in (A)].

To examine the possibility of such correlated evolution between labyrinthine and spot motifs in fish lineages, I applied phylogenetic comparative methods (*29*) to the genus-level trait data for each of the top 10 major fish orders: Cypriniformes, Siluriformes, Perciformes, Cichliformes, Characiformes, Gobiiformes, Cyprinodontiformes, Blenniiformes, Pleuronectiformes, and Tetraodontiformes (ranked based on the number of genera within each order). I tested independent and dependent models of pattern motif evolution (fig. S7): Under the independent model, all three motifs (Sp-D, Sp-L, and Maze) evolve independently without affecting one another, while under the dependent model, the evolution of one motif can be affected by the state of the other two motifs. Bayesian analyses (*30*) found substantial support for the dependent model (correlated evolution of labyrinthine and spot motifs) in 8 of 10 orders (log Bayes factor = 4.18 to 43.1; table S2). Furthermore, the estimated rates of transition from “without Maze” to “with Maze” (Maze gain rate, *a* to *d* in Fig. 6A) were higher when both dark and light spot motifs were included in the pattern portfolio (rate *d*), than when one or both of these spot motifs happened to be absent (rates *a* to *c*) (Fig. 6B). By contrast, transition rates from “with Maze” to “without Maze” (Maze loss rate, *e* to *h* in Fig. 6A) tended to be lower when both spot motifs coexisted (Fig. 6C). This means that the coexistence of dark and light spot motifs increases the probability of the emergence of labyrinthine motif and reduces the probability of its loss. These tendencies were also confirmed in further analyses, including comparisons with other motif combinations performed on the entire fish lineage (figs. S8 and S9). These findings suggest the frequent occurrence of pattern blending events in multiple major fish lineages and its contribution to the enrichment of color pattern diversity.

## DISCUSSION

In this study, I presented empirical evidence supporting the pattern blending hypothesis. Comprehensive analysis of pattern diversity among fish skins revealed strong mechanistic links for the qualitatively distinct pattern motifs, which are consistent with the hypothesis. Furthermore, the pufferfish examples clearly demonstrated that pattern blending actually occurs in the wild: Hybridization between species with simple spot patterns can create camouflaged, labyrinthine organisms that appear like distinct or novel species, just as the model predicted.

In nature, there are many species that have labyrinthine motifs. I found more than 900 fish species with labyrinthine motifs from >160 families, accounting for 5% of all fish species and 30% of all fish families examined here (Fig. 2A). It is reasonable to expect that at least a part of these labyrinthine animals originated from interspecific hybridization between spotted species, as is the case for *Arothron* pufferfishes. I anticipate that some of them may be just hybrids and have been deceiving taxonomists with their camouflaged coloration to be given unworthy taxonomic positions as novel/distinct species.

Another evolutionarily more intriguing possibility could be hybrid speciation (*31*). In the case of *Arothron* pufferfish, many traces of interspecific hybridization/introgression were detected between labyrinthine species and their spotted relatives. Moreover, in multiple major fish lineages, I found strong support for the correlated evolution of labyrinthine and spot motifs: Coexistence of dark and light spot species substantially increased the probability of the emergence of a labyrinthine motif, suggesting the frequent occurrence of pattern blending by hybridization and consequential hybrid speciation.

Accumulating evidence now indicates that hybridization is a potential driver of biological diversity (*13*, *28*, *31*–*35*). Recently, it was shown that interspecific hybridization can form a new species very rapidly, even within a few generations (*36*). Considering that animal markings are frequently used as visual cues for the recognition of a species and mate choice (*5*, *6*), qualitative and drastic changes in color patterns from parent species may easily result in reproductive isolation under conditions of assortative mating (*37*, *38*). In addition, a conspicuous yet disguising appearance can have multiple advantages in certain circumstances: camouflage from predators/prey by means of background matching or disruptive coloration, and aposematic signals if present with strong defensive tools, such as poisons or spines (*1*–*3*). Thus, both of the two major requirements for hybrid speciation, namely, reproductive isolation from the parent species and exploitation of new niches (*31*), can be fulfilled with regard to the camouflaged labyrinthine color patterns.

Although I do not rule out any other evolutionary, developmental, and ecological factors that may have caused camouflaged colorations to arise, it could be evolutionary feasible that a group of hybrid individuals with “intermediate and transgressive” phenotypes, such as labyrinthine patterns, evolved as a distinct lineage of hybrid origin to form a new species [speciation by fusion (*38*)]. I expect that such reticulate evolution of color patterns may account for a key part of the general pattern diversity―ubiquitous, long-range similarity over local divergence―observed among animals. Further exploration of diverse biological groups, including rare species in museum collections, should shed light on the rich structure of the phenotypic space for this seemingly two-dimensional trait, and possibly uncover a secret path through the evolutionary labyrinth of animal color patterns.

## MATERIALS AND METHODS

### Mathematical modeling and simulation

Mathematical modeling and numerical simulation were performed as previously described (*18*). Briefly, I used a class of RD system described as

$$\begin{array}{c} \frac{\partial u}{\partial t}=\mathrm{Rf}(u,v)+D_{u}\nabla^{2}u\\ \frac{\partial v}{\partial t}=\mathrm{Rg}(u,v)+D_{v}\nabla^{2}v \end{array}$$

where *u* and *v* are the concentrations of hypothetical factors, *f* and *g* are the reaction kinetics, and *D_u_* and *D_v_* are the hypothetical diffusion coefficients (or their mathematical equivalents) for *u* and *v*, respectively. The reaction rate, *R*, was introduced for the convenience of parameter adjustment. The reaction kinetics and parameters are as follows

$$\begin{array}{c} f(u,v)=(\mathrm{Au}+\mathrm{Bv}+C)-\mathrm{Du}\\ g(u,v)=(\mathrm{Eu}-F)-\mathrm{Gv} \end{array}$$

where *A* = 0.08, *B* = 0.08, *D* = 0.03, *E* = 0.1, *F* = 0.12, *G* = 0.06, *D_u_* = 0.5, *D_v_* = 10.0, and *R* = 80 unless otherwise indicated, and the lower and upper limits for the synthesis rates of *u* (*Au* + *Bv* + *C*) and *v* (*Eu* – *F*) are set as

$$\begin{array}{c} 0\leq (\mathrm{Au}+\mathrm{Bv}+C)\leq \text{syn}\;U_{\text{max}}\\ 0\leq (\mathrm{Eu}-F)\leq \text{syn}\;V_{\text{max}} \end{array}$$

where syn*U*_max_ = 0.23 and syn*V*_max_ = 0.5.

Simulations were carried out on 25,000 to 55,000 evenly distributed cells on spheres or fused blobs (metaballs). The coordinates of each cell are arbitrarily given in the simulations. In the simulations on fused blobs, a different set of parameter values can be set for each cell. Those values are determined by the position of each cell: Cells at each end of the blob have different extreme parameter values, while cells in the central fused region have gradual intermediate parameter values, representing the result of in silico “hybridization.” The initial conditions included random patterns of *u* and *v*. The time step for all simulations was set as *dt* = 0.01. Calculations were performed with 2000 iterations. The POV-Ray software and Vapory Python library were used for rendering. Note that similar pattern transitions to those observed in these simulations can be reproduced using a variety of other models (*39*, *40*).

### Annotation of fish color patterns

Fish images with identified species names were referenced from public databases (FishBase: https://www.fishbase.org/ and FishPix: http://fishpix.kahaku.go.jp/fishimage-e/). A total of 19,755 images of 18,114 species from 4069 genera and 559 families were subjected to pattern annotation using a web-based in-house annotation system. Based on the descriptions of pattern motifs commonly used in original papers, pictorial books, and other publications on this topic (*7*, *8*), I formulated the following 11 classes of pattern motifs: 4 types of stripe patterns (St-H, horizontal or longitudinal stripes running parallel to the body axis; St-D, diagonal stripes/bands running at an angle of roughly 20° to 70° to the body axis; St-V, vertical stripes/bars oriented perpendicular to the body axis; and Maze, labyrinthine stripes with less or no directional anisotropy), 3 types of spot patterns (Sp-D, small dark spots that are evenly distributed on a light background; Sp-L, small light spots on a dark background; and Eyes, ocellated spots or one or a few spots larger than the actual eyes), and 4 other patterns (Area, area fill pattern in which large areas are painted in a single color; Sddl, saddle-like markings that line up on the back of the body; Bltc, blotch/speckle pattern with irregularly shaped and not evenly spaced markings; and Mono, monotone/patternless). Pattern annotation was carried out by multilabel classification, that is, each image was binary labeled for each class of pattern motifs (1, containing the pattern motif; 0, not containing the pattern motif).

### Co-occurrence analysis of color pattern variations

For each genus and each class of pattern motifs, the occurrence of species having that pattern motif was counted and binary labeled (1, containing species with the pattern motif; 0, not containing species with the pattern motif) (fig. S2A). Next, within-genus pattern co-occurrence between each pairwise combination of pattern classes was examined and counted (fig. S2B). The significance and strength of the pattern motif co-occurrences were evaluated using six association measures (*21*, *22*): JI, SDC, SSI, LL, *T* score (*T*), and *z* score (*Z*). The association measures for the motif A and B pair are defined as

$$\begin{array}{c} \text{JI}=\frac{O_{11}}{O_{10}+O_{01}+O_{11}}\\ \text{SDC}=\frac{2O_{11}}{A_{1}+B_{1}}\\ \text{SSI}=\frac{O_{11}}{\text{min}(A_{1},B_{1})}\\ \text{LL}=2\underset{\mathrm{ij}}{\Sigma }O_{\mathrm{ij}}\text{log}\frac{O_{\mathrm{ij}}}{E_{\mathrm{ij}}}\\ T=\frac{O_{11}-E_{11}}{\sqrt{O_{11}}}\\ Z=\frac{O_{11}-E_{11}}{\sqrt{E_{11}}} \end{array}$$

where *O_ij_* is the observed frequencies, *i* and *j* denote occurrence (1) or nonoccurrence (0) of motifs A and B, respectively (e.g., *O*_11_ is the number of genera where motifs A and B co-occur, whereas *O*_10_ is the number of genera where motif A occurs but motif B does not), *A_i_* and *B_j_* are the total numbers of occurrence (*i*, *j* = 1) or nonoccurrence (*i*, *j* = 0) of motifs A and B, respectively, and *E_ij_* = *A_i_B_j_*/Σ*_ij_O_ij_* is the expected frequencies. A total of 2384 genera containing two or more species was used for the calculation. For the calculation of association measures for triple co-occurrence, a total of 1651 genera containing three or more species was used, and a combination of two motifs [e.g., dark (Sp-D) and light (Sp-L) spots] was treated as motif A in the above definition (i.e., the occurrence of motif A means the co-occurrence of Sp-D and Sp-L; fig. S2C).

### Quantitative analysis of color patterns

Various methods and metrics have been so far developed and proposed for the quantification of color patterns (*18*, *41*, *42*). For a detailed quantitative analysis of the body patterns of *Arothron* species, I focused on two indicators: pattern complexity (based on element-wise circularity/elongation) and overall color tone (pattern lightness measured as the proportion of unpigmented area in a binarized image) (*18*). The pattern complexity score (PCS) is defined based on the area-weighted mean isoperimetric quotient of the contours extracted from each image

$$\text{PCS}=1-\underset{i}{\Sigma }w_{i}Q_{i}$$

where *Q_i_* = 4π*S_i_*/*L_i_*^2^ is the isoperimetric quotient (or circularity) of each contour, *w_i_* = *S_i_*/Σ*_i_S_i_* is the area weight, and *S_i_* and *L_i_* are the area and perimeter of each contour, respectively. Here, pattern complexity was calculated on the basis of the area and contour length for each pattern element that makes up the overall pattern. The area of a shape enclosed by a contour is minimized when the shape takes its simplest form, a circle. As the shape becomes more complex, the contour becomes longer. Therefore, as a measure of the complexity of a shape, we can use the “circularity” of the shape, which is calculated by comparing the area of the shape enclosed by a contour of a certain length with the area of a circle with a circumference of the same length. The circularity takes a maximum value of 1 for a circle (the simplest shape) and decreases as the shape deviates from the circle. The complexity of the entire pattern can be calculated from this circularity obtained for each element. However, using a simple average of the element-wise circularity may be inappropriate because the overall impression of the complexity of a pattern depends on the pattern elements that occupy a large area of the entire pattern. For example, when a region of interest contains one complex, dominantly large element and a large number of simple, small elements, the pattern complexity calculated from the simple average of the element-wise circularity may underestimate the overall complexity of that region. Therefore, in this study, I used an area-weighted average of circularity and calculated the PCS by subtracting this value from 1. These quantification analyses were performed on binarized images using the OpenCV library and in-house Python script.

The PCS used in this study is considered appropriate as a quantitative index that can reliably capture the characteristics of patterns such as spots and maze and the transitions between them (Fig. 4); however, it is difficult to measure the differences in complexity between other combinations of patterns such as horizontal or vertical stripes and maze. For the latter, it may be useful to use other indices such as the aspect ratio and orientation of each pattern element (*41*) and/or their variances.

### Sampling, DNA extraction, library preparation, and sequencing

A total of 21 individual/tissue specimens from 12 *Arothron* species were obtained from various sources, including museum/aquarium collections and local aquarium shops. Identification of the species and nomenclature followed Matsuura (*23*, *24*). Total DNA was extracted from tissue samples of pectoral fin and/or skeletal muscle using the DNeasy Blood & Tissue Kit (Qiagen, 69504). Double-stranded genomic DNA libraries for high-throughput sequencing were prepared either with the TruSeq DNA PCR-Free/Nano DNA Library Prep Kit (Illumina, FC-121-3001/4001) or the NEBNext Ultra II DNA Library Prep Kit (New England Biolabs, E7645S).

Because the two labyrinthine species, *A. carduus* and *A. multilineatus*, are extremely rare, I used formalin-fixed specimens deposited in museum collections (HUMZ 35438 and KAUM-I. 13606, collected in 1973 and 2009, respectively). For the extraction of very short, degraded DNA fragments [mode length, 30 to 40 base pairs (bp)] from these museum specimens, the DNAs-ici!-F (Rizo, DS-0005) was used. In addition to the double-stranded DNA libraries, single-stranded DNA libraries were constructed for museum samples, based on the methods developed for ancient DNA analysis (*43*) with the following modifications: (i) in the extension step (steps 12 to 14), the extension primer CL9 was replaced with a newly designed extension primer for P5 (EPP5: 5′-ACACTCTTTCCCTACACGACGCTCTTCCGATCT-3′), so that widely used universal primers could be used for easy outsourcing of DNA sequencing, (ii) the concentration of 2′-deoxyadenosine 5′-triphosphate (dATP) was increased by fivefold to promote the generation of 3′ adenine overhangs, so that (iii) the blunt-end repair steps (steps 16 to 19) could be omitted, and the second adaptor ligation steps (steps 20 to 22) could be performed using the Ligation Module in NEBNext Ultra II DNA Library Prep Kit (New England Biolabs) and the NEBNext Adaptors. To avoid potential contamination, DNA extraction and library preparation for formalin-fixed museum specimens were conducted in an isolated room with a clean bench.

Whole-genome sequencing was performed at Macrogen using the Illumina HiSeq X platform. All animal experiments were conducted in accordance with the institutional guidelines of Osaka University.

### Assembly of mitochondrial genomes and phylogenetic analysis

Reads were trimmed with Trimmomatic v0.36 (*44*) and mapped to a published whole mitochondrial genome of *A. firmamentum* (AP006742) using BWA v0.7.15 (*45*) (BWA-MEM) with the default options. Complete mitochondrial genome assembly for each sample was reconstructed using freebayes v1.1.0-46-g8d2b3a0 (*46*) and bcftools v1.8 (bcftools consensus), complemented with Sanger sequencing for the D-loop regions. For the museum samples, reads from single- and double-stranded libraries were trimmed and collapsed with AdapterRemoval v2.2.2 (*47*) and mapped to each of the above assemblies using BWA (BWA-backtrack: bwa aln -n 5 -o 1 -l 16500; bwa samse). After the removal of reads that were too short (<28 bp), too long (>60 bp), duplicated, or spuriously aligned, nearly complete mitochondrial genome assemblies were built by consensus calling using Analysis of Next Generation Sequencing Data (ANGSD) v0.918 (*48*) (angsd -doFasta 2).

A total of 12 mitochondrial genome assemblies were annotated using MitoAnnotator (*49*) with some manual corrections. Annotated sequences and two published outgroup mitogenomes (NC_010979: *Canthigaster rivulata*, NC_031325: *Dichotomyctere nigroviridis*) were multiple aligned and trimmed using Mafft v7.313 (*50*) and trimal v1.4 (*51*), respectively. Five partitions were set and subjected to maximum likelihood analysis using RAxML v8.2.11 (*52*) (rapid bootstrap analysis with 1000 replications). The general time reversible + gamma model (GTR + GAMMA) was selected using Kakusan4 v4.0.2016.11.04 (*53*) based on Akaike information criterion.

### De novo whole-genome assembly, mapping, and estimation of genotype likelihood

Initial attempts to map the reads to the publicly available reference genomes of other pufferfish species (*Takifugu rubripes* and *D. nigroviridis*) resulted in low mapping rates (up to 44 and 74%, respectively); therefore, I generated a de novo assembly of one *Arothron* species (*A. firmamentum*) using Platanus v1.2.4 (*54*). Trimmed reads (insert size, 350 bp) were entered into Platanus, and gap-closed scaffolds were constructed using default options. After discarding <500-bp sequences, a draft genome spanning 335 Mb was obtained (aroFir_1.0: 10,793 scaffolds; N50 scaffold size, 135 kb). For assessment of the quality of the genome assembly, BUSCO v3.0.2 (*55*) was used with the actinopterygii_odb9 dataset (complete, 94.9% [single copy: 92.5%, duplicated: 2.4%]; fragmented, 2.5%; missing, 2.6%, *n* = 4584 BUSCOs). Trimmed reads of each *Arothron* sample were mapped to this reference genome using BWA-MEM with the default options, and then duplicated reads were removed with MarkDuplicates in Picard tools v.2.11.0. For museum samples, trimmed and collapsed reads were mapped using BWA-backtrack (bwa aln -n 0.01 -o 2 -l 16500), after which duplicated reads were removed with the rmdup_collapsed command in PALEOMIX v1.2.12 (*56*). The output bam files were subsampled to at most 30× and processed with ANGSD for genotype likelihood estimation (angsd -GL 2 -doGlf 2 -doMajorMinor 1 -doMaf 1 -SNP_pval 2e-6 -minMapQ 30 -minQ 20 -minInd 19).

### Inferring population structure, admixture proportions, and introgression

PCA was performed on the output file from ANGSD in Beagle format using PCAngsd v0.95 (*57*), which can handle genotype likelihoods as input data for improved accuracy in samples with low and variable sequencing depth. An in-house Python script was used for plotting. Genome-wide admixture proportions for each individual were estimated using NGSadmix v32 (*58*), with *K* = 4 to 6 ancestral populations based on the same dataset.

Interclass heterozygosity (the proportion of loci with alleles from both ancestral populations) of possible hybrid individuals were estimated using ANGSD and an in-house script based on a total of 5,198,905 diagnostic biallelic sites that segregate five individuals each of *A. reticularis* and *A. stellatus*.

ABBA-BABA analysis was performed using the *D. nigroviridis* genome (tetNig2) as an outgroup and reference; reads from *Arothron* species were remapped to the tetNig2 reference genome using BWA-MEM or BWA-backtrack (options are the same as those used for the aroFir_1.0 reference), and then processed with ANGSD (angsd -doAbbababa 1 -doCounts 1 -blockSize 5000000 -anc tetNig2.fa). Patterson’s *D* statistic (*27*) for each combination of *Arothron* species was calculated, and the significance was evaluated with *z* scores obtained by the block-jackknifing using 5-Mb blocks. The *D*_min_ statistic (*28*) was obtained as the minimum absolute value of Patterson’s *D* for each trio of *Arothron* species across all possible tree topologies *D*_min_ = min {∣*D*(*A*, *B*; *C*, *O*)∣, ∣*D*(*A*, *C*; *B*, *O*)∣, ∣*D*(*C*, *B*; *A*, *O*)∣}.

### Analyses of correlated evolution of pattern motifs

For the phylogenetic comparative analyses, I used the time-calibrated phylogeny of ray-finned fishes (*59*) obtained from https://fishtreeoflife.org by using the R package “fishtree” (*60*). I pruned the tree by randomly selecting a single representative species per each genus for which genus-level pattern annotation data were available and prepared genus-level tree samples for each order by repeating this process 500 times per order.

Analyses of correlated evolution among pattern motifs were performed using BayesTraits V3.0.1 (*30*). I defined the portfolio of pattern motifs in a genus as the trait of that genus. The combinations of the presence/absence of each of the three pattern motifs (Sp-D, Sp-L, and Maze) were coded into eight different states (i.e., 000, 100, …, 111), and the Multistate module with reversible-jump Markov chain Monte Carlo (MCMC) in BayesTraits was used to estimate the evolutionary transition rates among these states for each order. I tested independent (IND) and dependent (DEP) models of pattern motif evolution (fig. S7): Under the independent model, all three motifs evolve independently without affecting one another, while under the dependent model, the evolution of one motif can be affected by the state of the other two motifs. I used a uniform hyperprior ranging from 0 to 10 to seed the mean of the exponential prior. Each MCMC analysis was run three times for 600 million iterations with a burn-in of 100 million and sampling every 50,000 iterations. The marginal likelihood was estimated by the stepping stone sampler with 100 stones and 10,000 iterations per stone. Log Bayes factors were calculated as twice the difference between the log marginal likelihood of the dependent and independent models. I took log Bayes factors >2 as positive evidence, >5 as strong evidence, and >10 as very strong evidence (*30*).

Further analyses were performed on the entire fish lineage (2837 genera for which trait data were available) and various sets of three motif combinations. I tested partially independent models (ind*X*, ind*Y*, and ind*Z*), in which two motifs are correlated (evolve dependently), while the evolution of the other motif is independent (figs. S8A and S9A), as well as the independent and dependent models described above, for the following sets of three motif combinations: (i) dark spots, light spots, and one of the other nine motifs (Sp-D, Sp-L, and motif *Z*) (fig. S8, B and C), (ii) horizontal and vertical stripes and labyrinthine motif (St-H, St-V, and Maze), and one stripe motif, one spot motif, and labyrinthine motif (St-H/St-V, Sp-D/Sp-L, and Maze) (fig. S9, B and C). For these analyses, I used the same hyperprior as above and performed three MCMC runs each for 6 million iterations with a burn-in of 1 million and sampling every 5000 iterations. The marginal likelihood was estimated by the stepping stone sampler with 100 stones and 1000 iterations per stone. Log Bayes factors were calculated as twice the difference between the log marginal likelihood of each model.

## Supplementary Material

http://advances.sciencemag.org/cgi/content/full/6/49/eabb9107/DC1

Data file S1

Adobe PDF - abb9107_SM.pdf

Pattern blending enriches the diversity of animal colorations

## SUPPLEMENTARY MATERIALS

Supplementary material for this article is available at http://advances.sciencemag.org/cgi/content/full/6/49/eabb9107/DC1

## Appendix

View/request a protocol for this paper from *Bio-protocol*.

## References

[1] Wallace A. R., The colors of animals and plants. Am. Nat. 11, 641–662 (1877).

[2] Cuthill I. C., Allen W. L., Arbuckle K., Caspers B., Chaplin G., Hauber M. E., Hill G. E., Jablonski N. G., Jiggins C. D., Kelber A., Mappes J., Marshall J., Merrill R., Osorio D., Prum R., Roberts N. W., Roulin A., Rowland H. M., Sherratt T. N., Skelhorn J., Speed M. P., Stevens M., Stoddard M. C., Stuart-Fox D., Talas L., Tibbetts E., Caro T., The biology of color. Science 357, eaan0221 (2017). 28774901 10.1126/science.aan0221

[3] M. Stevens, S. Merilaita, *Animal Camouflage: Mechanisms and Function* (Cambridge Univ. Press, Cambridge, UK, 2011).

[4] Caro T., Izzo A., Reiner R. C. Jr., Walker H., Stankowich T., The function of zebra stripes. Nat. Commun. 5, 3535 (2014). 24691390 10.1038/ncomms4535

[5] Houde A. E., Endler J. A., Correlated evolution of female mating preferences and male color patterns in the guppy *Poecilia reticulata*. Science 248, 1405–1408 (1990). 17747527 10.1126/science.248.4961.1405

[6] Seehausen O., Terai Y., Magalhaes I. S., Carleton K. L., Mrosso H. D. J., Miyagi R., van der Sluijs I., Schneider M. V., Maan M. E., Tachida H., Imai H., Okada N., Speciation through sensory drive in cichlid fish. Nature 455, 620–626 (2008). 18833272 10.1038/nature07285

[7] T. Nakabo, *Fishes of Japan: With Pictorial Keys to the Species* (Tokai Univ. Press, Kanagawa, Japan, ed. 3, 2013).

[8] G. Allen, R. Steel, P. Humann, N. Deloach, *Reef Fish Identification: Tropical Pacific* (New World Publications Inc., Jacksonville, FL, ed. 2, 2015).

[9] Singh A. P., Nüsslein-Volhard C., Zebrafish stripes as a model for vertebrate colour pattern formation. Curr. Biol. 25, R81–R92 (2015). 25602311 10.1016/j.cub.2014.11.013

[10] Patterson L. B., Parichy D. M., Zebrafish pigment pattern formation: Insights into the development and evolution of adult form. Annu. Rev. Genet. 53, 505–530 (2019). 31509458 10.1146/annurev-genet-112618-043741

[11] Yamanoue Y., Miya M., Matsuura K., Miyazawa S., Tsukamoto N., Doi H., Takahashi H., Mabuchi K., Nishida M., Sakai H., Explosive speciation of *Takifugu*: Another use of fugu as a model system for evolutionary biology. Mol. Biol. Evol. 26, 623–629 (2009). 19074759 10.1093/molbev/msn283

[12] Werner T., Koshikawa S., Williams T. M., Carroll S. B., Generation of a novel wing colour pattern by the Wingless morphogen. Nature 464, 1143–1148 (2010). 20376004 10.1038/nature08896

[13] The Heliconius Genome Consortium, Butterfly genome reveals promiscuous exchange of mimicry adaptations among species. Nature 487, 94–98 (2012). 22722851 10.1038/nature11041 PMC3398145

[14] Mallarino R., Henegar C., Mirasierra M., Manceau M., Schradin C., Vallejo M., Beronja S., Barsh G. S., Hoekstra H. E., Developmental mechanisms of stripe patterns in rodents. Nature 539, 518–523 (2016). 27806375 10.1038/nature20109 PMC5292240

[15] Kratochwil C. F., Liang Y., Gerwin J., Woltering J. M., Urban S., Henning F., Machado-Schiaffino G., Hulsey C. D., Meyer A., Agouti-related peptide 2 facilitates convergent evolution of stripe patterns across cichlid fish radiations. Science 362, 457–460 (2018). 30361373 10.1126/science.aao6809

[16] Kaelin C. B., Xu X., Hong L. Z., David V. A., McGowan K. A., Schmidt-Küntzel A., Roelke M. E., Pino J., Pontius J., Cooper G. M., Manuel H., Swanson W. F., Marker L., Harper C. K., van Dyk A., Yue B., Mullikin J. C., Warren W. C., Eizirik E., Kos L., O’Brien S. J., Barsh G. S., Menotti-Raymond M., Specifying and sustaining pigmentation patterns in domestic and wild cats. Science 337, 1536–1541 (2012). 22997338 10.1126/science.1220893 PMC3709578

[17] Haupaix N., Curantz C., Bailleul R., Beck S., Robic A., Manceau M., The periodic coloration in birds forms through a prepattern of somite origin. Science 361, eaar4777 (2018). 30237324 10.1126/science.aar4777

[18] Miyazawa S., Okamoto M., Kondo S., Blending of animal colour patterns by hybridization. Nat. Commun. 1, 66 (2010). 20842190 10.1038/ncomms1071 PMC2982180

[19] Kondo S., Miura T., Reaction-diffusion model as a framework for understanding biological pattern formation. Science 329, 1616–1620 (2010). 20929839 10.1126/science.1179047

[20] Turing A. M., The chemical basis of morphogenesis. Philos. Trans. R. Soc. Lond. B. 237, 37–72 (1952).

[21] A. E. Magurran, *Measuring Biological Diversity* (Blackwell Science Ltd., Malden, MA, 2004).

[22] S. Hoffmann, S. Evert, N. Smith, D. Lee, Y. Berglund Prytz, *Corpus Linguistics with BNCweb - a Practical Guide* (Peter Lang GmbH, Frankfurt am Main, Germany, 2008).

[23] K. Matsuura, *Pufferfishes and their Allies in Japan* (Tokai Univ. Press, Kanagawa, Japan, 2017).

[24] Matsuura K., A new pufferfish, *Arothron multilineatus* (Actinopterygii: Tetraodontiformes: Tetraodontidae), from the Indo-West Pacific. Ichthyol. Res. 63, 480–486 (2016).

[25] Fitzpatrick B. M., Estimating ancestry and heterozygosity of hybrids using molecular markers. BMC Evol. Biol. 12, 131 (2012). 22849298 10.1186/1471-2148-12-131 PMC3572440

[26] Cantor T. E., Catalogue of Malayan fishes. J. Asiat. Soc. Bengal. 18, 981–1443 (1849).

[27] Green R. E., Krause J., Briggs A. W., Maricic T., Stenzel U., Kircher M., Patterson N., Li H., Zhai W., Fritz M. H.-Y., Hansen N. F., Durand E. Y., Malaspinas A.-S., Jensen J. D., Marques-Bonet T., Alkan C., Prüfer K., Meyer M., Burbano H. A., Good J. M., Schultz R., Aximu-Petri A., Butthof A., Höber B., Höffner B., Siegemund M., Weihmann A., Nusbaum C., Lander E. S., Russ C., Novod N., Affourtit J., Egholm M., Verna C., Rudan P., Brajkovic D., Kucan Ž., Gušic I., Doronichev V. B., Golovanova L. V., Lalueza-Fox C., de la Rasilla M., Fortea J., Rosas A., Schmitz R. W., Johnson P. L. F., Eichler E. E., Falush D., Birney E., Mullikin J. C., Slatkin M., Nielsen R., Kelso J., Lachmann M., Reich D., Pääbo S., A draft sequence of the Neandertal genome. Science 328, 710–722 (2010). 20448178 10.1126/science.1188021 PMC5100745

[28] Malinsky M., Svardal H., Tyers A. M., Miska E. A., Genner M. J., Turner G. F., Durbin R., Whole-genome sequences of Malawi cichlids reveal multiple radiations interconnected by gene flow. Nat. Ecol. Evol. 2, 1940–1955 (2018). 30455444 10.1038/s41559-018-0717-x PMC6443041

[29] Pagel M., Detecting correlated evolution on phylogenies: A general method for the comparative analysis of discrete characters. Proc. R. Soc. B Biol. Sci. 255, 37–45 (1994).

[30] Pagel M., Meade A., Bayesian analysis of correlated evolution of discrete characters by reversible-jump Markov chain Monte Carlo. Am. Nat. 167, 808–825 (2006). 16685633 10.1086/503444

[31] Mallet J., Hybrid speciation. Nature 446, 279–283 (2007). 17361174 10.1038/nature05706

[32] Mavárez J., Salazar C. A., Bermingham E., Salcedo C., Jiggins C. D., Linares M., Speciation by hybridization in *Heliconius* butterflies. Nature 441, 868–871 (2006). 16778888 10.1038/nature04738

[33] Meier J. I., Marques D. A., Mwaiko S., Wagner C. E., Excoffier L., Seehausen O., Ancient hybridization fuels rapid cichlid fish adaptive radiations. Nat. Commun. 8, 14363 (2017). 28186104 10.1038/ncomms14363 PMC5309898

[34] Irisarri I., Singh P., Koblmüller S., Torres-Dowdall J., Henning F., Franchini P., Fischer C., Lemmon A. R., Lemmon E. M., Thallinger G. G., Sturmbauer C., Meyer A., Phylogenomics uncovers early hybridization and adaptive loci shaping the radiation of Lake Tanganyika cichlid fishes. Nat. Commun. 9, 3159 (2018). 30089797 10.1038/s41467-018-05479-9 PMC6082878

[35] Jones M. R., Mills L. S., Alves P. C., Callahan C. M., Alves J. M., Lafferty D. J. R., Jiggins F. M., Jensen J. D., Melo-Ferreira J., Good J. M., Adaptive introgression underlies polymorphic seasonal camouflage in snowshoe hares. Science 1358, 1355–1358 (2018). 10.1126/science.aar5273 29930138

[36] Lamichhaney S., Han F., Webster M. T., Andersson L., Grant B. R., Grant P. R., Rapid hybrid speciation in Darwin’s finches. Science 359, 224–228 (2018). 29170277 10.1126/science.aao4593

[37] Jiang Y., Bolnick D. I., Kirkpatrick M., Assortative mating in animals. Am. Nat. 181, E125–E138 (2013). 23669548 10.1086/670160

[38] Grant P. R., Grant B. R., Role of sexual imprinting in assortative mating and premating isolation in Darwin’s finches. Proc. Natl. Acad. Sci. U.S.A. 115, E10879–E10887 (2018). 30348758 10.1073/pnas.1813662115 PMC6243256

[39] J. D. Murray, *Mathematical Biology* (Springer-Verlag, New York, ed. 3, 2002).

[40] Gowda K., Chen Y., Iams S., Silber M., Assessing the robustness of spatial pattern sequences in a dryland vegetation model. Proc. R. Soc. A Math. Phys. Eng. Sci. 472, 20150893 (2016). 10.1098/rspa.2015.0893 PMC4841491 27118924

[41] Chan I. Z. W., Stevens M., Todd P. A., PAT-GEOM: A software package for the analysis of animal patterns. Methods Ecol. Evol. 10, 591–600 (2019).

[42] Van Belleghem S. M., Papa R., Ortiz-Zuazaga H., Hendrickx F., Jiggins C. D., Owen McMillan W., Counterman B. A., patternize: An R package for quantifying colour pattern variation. Methods Ecol. Evol. 9, 390–398 (2018). 29755717 10.1111/2041-210X.12853 PMC5945207

[43] Gansauge M. T., Meyer M., Single-stranded DNA library preparation for the sequencing of ancient or damaged DNA. Nat. Protoc. 8, 737–748 (2013). 23493070 10.1038/nprot.2013.038

[44] Bolger A. M., Lohse M., Usadel B., Trimmomatic: A flexible trimmer for Illumina sequence data. Bioinformatics 30, 2114–2120 (2014). 24695404 10.1093/bioinformatics/btu170 PMC4103590

[45] H. Li, Aligning sequence reads, clone sequences and assembly contigs with BWA-MEM. arXiv:1303.3997 [q-bio.GN] (2013).

[46] E. Garrison, G. Marth, Haplotype-based variant detection from short-read sequencing. arXiv:1207.3907 [q-bio.GN] (2012).

[47] Schubert M., Lindgreen S., Orlando L., AdapterRemoval v2: Rapid adapter trimming, identification, and read merging. BMC. Res. Notes 9, 88 (2016). 26868221 10.1186/s13104-016-1900-2 PMC4751634

[48] Korneliussen T. S., Albrechtsen A., Nielsen R., ANGSD: Analysis of next generation sequencing data. BMC Bioinformatics. 15, 356 (2014). 25420514 10.1186/s12859-014-0356-4 PMC4248462

[49] Iwasaki W., Fukunaga T., Isagozawa R., Yamada K., Maeda Y., Satoh T. P., Sado T., Mabuchi K., Takeshima H., Miya M., Nishida M., Mitofish and mitoannotator: A mitochondrial genome database of fish with an accurate and automatic annotation pipeline. Mol. Biol. Evol. 30, 2531–2540 (2013). 23955518 10.1093/molbev/mst141 PMC3808866

[50] Katoh K., Standley D. M., MAFFT multiple sequence alignment software version 7: Improvements in performance and usability. Mol. Biol. Evol. 30, 772–780 (2013). 23329690 10.1093/molbev/mst010 PMC3603318

[51] Capella-gutiérrez S., Silla-martínez J. M., Gabaldón T., trimAl : A tool for automated alignment trimming in large-scale phylogenetic analyses. Bioinformatics 25, 1972–1973 (2009). 19505945 10.1093/bioinformatics/btp348 PMC2712344

[52] Stamatakis A., RAxML version 8: A tool for phylogenetic analysis and post-analysis of large phylogenies. Bioinformatics 30, 1312–1313 (2014). 24451623 10.1093/bioinformatics/btu033 PMC3998144

[53] Tanabe A. S., Kakusan4 and Aminosan: Two programs for comparing nonpartitioned, proportional and separate models for combined molecular phylogenetic analyses of multilocus sequence data. Mol. Ecol. Resour. 11, 914–921 (2011). 21592310 10.1111/j.1755-0998.2011.03021.x

[54] Kajitani R., Toshimoto K., Noguchi H., Toyoda A., Ogura Y., Okuno M., Yabana M., Harada M., Nagayasu E., Maruyama H., Kohara Y., Fujiyama A., Hayashi T., Itoh T., Efficient de novo assembly of highly heterozygous genomes from whole-genome shotgun short reads. Genome Res. 24, 1384–1395 (2014). 24755901 10.1101/gr.170720.113 PMC4120091

[55] Waterhouse R. M., Seppey M., Simão F. A., Manni M., Ioannidis P., Klioutchnikov G., Kriventseva E. V., Zdobnov E. M., BUSCO applications from quality assessments to gene prediction and phylogenomics. Mol. Biol. Evol. 35, 543–548 (2018). 29220515 10.1093/molbev/msx319 PMC5850278

[56] Schubert M., Ermini L., Sarkissian C. D., Jónsson H., Ginolhac A., Schaefer R., Martin M. D., Fernández R., Kircher M., Cue M. M., Willerslev E., Orlando L., Characterization of ancient and modern genomes by SNP detection and phylogenomic and metagenomic analysis using PALEOMIX. Nat. Protoc. 9, 1056–1082 (2014).24722405

[57] Meisner J., Albrechtsen A., Inferring population structure and admixture proportions in low-depth NGS data. Genetics 210, 719–731 (2018). 30131346 10.1534/genetics.118.301336 PMC6216594

[58] Skotte L., Korneliussen T. S., Albrechtsen A., Estimating individual admixture proportions from next generation sequencing data. Genetics 195, 693–702 (2013). 24026093 10.1534/genetics.113.154138 PMC3813857

[59] Rabosky D. L., Chang J., Title P. O., Cowman P. F., Sallan L., Friedman M., Kaschner K., Garilao C., Near T. J., Coll M., Alfaro M. E., An inverse latitudinal gradient in speciation rate for marine fishes. Nature 559, 392–395 (2018). 29973726 10.1038/s41586-018-0273-1

[60] Chang J., Rabosky D. L., Smith S. A., Alfaro M. E., An R package and online resource for macroevolutionary studies using the ray-finned fish tree of life. Methods Ecol. Evol. 10, 1118–1124 (2019).

[61] Betancur-R R., Wiley E. O., Arratia G., Acero A., Bailly N., Miya M., Lecointre G., Ortí G., Phylogenetic classification of bony fishes. BMC Evol. Biol. 17, 162 (2017). 28683774 10.1186/s12862-017-0958-3 PMC5501477

