# Supplementary material for: Pattern blending enriches the diversity of animal colorations
Source: Sci Adv. 2020 Dec 2;6(49):eabb9107. doi: 10.1126/sciadv.abb9107 (PMC7710386; doi:10.1126/sciadv.abb9107)
Supplement: http://advances.sciencemag.org/cgi/content/full/6/49/eabb9107/DC1 [file supp_6_49_eabb9107__index.html]

Science Advances | Science AdvancesAAASSearchScience AdvancesMenu

## Supplementary Materials

# Pattern blending enriches the diversity of animal colorations

Seita Miyazawa

Download Supplement

**The PDF file includes:**

- Figs. S1 to S9
- Tables S1 and S2
- Legend for data file S1

**Other Supplementary Material for this manuscript includes the following:**

- Data file S1

**Files in this Data Supplement:**

- Adobe PDF - abb9107\_SM.pdf
