# Supplementary material for: Pattern blending enriches the diversity of animal colorations
Source: Sci Adv. 2020 Dec 2;6(49):eabb9107. doi: 10.1126/sciadv.abb9107 (PMC7710386; doi:10.1126/sciadv.abb9107)
Supplement: http://advances.sciencemag.org/cgi/content/full/6/49/eabb9107/DC1 [file supp_6_49_eabb9107__1.pdf]

Science Advances
AAAS

advances.sciencemag.org/cgi/content/full/6/49/eabb9107/DC1

Supplementary Materials for

Pattern blending enriches the diversity of animal colorations

Seita Miyazawa*

*Corresponding author. Email: seita@fbs.osaka-u.ac.jp

Published 2 December 2020, *Sci. Adv.* **6**, eabb9107 (2020)
DOI: 10.1126/sciadv.abb9107

The PDF file includes:

Figs. S1 to S9
Tables S1 and S2
Legend for data file S1

Other Supplementary Material for this manuscript includes the following:

(available at advances.sciencemag.org/cgi/content/full/6/49/eabb9107/DC1)

Data file S1

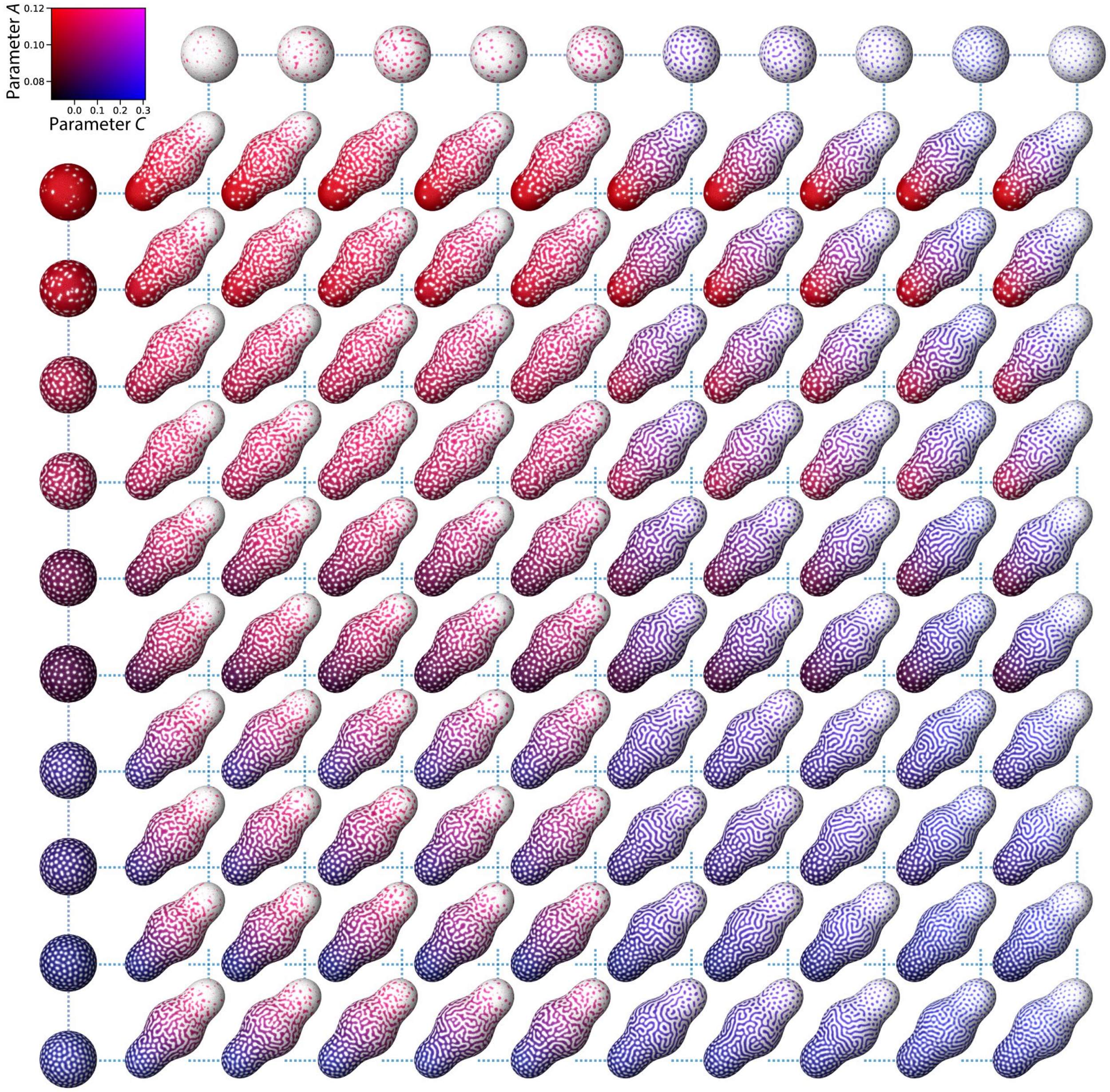

Fig. S1. Pattern variations generated by in silico hybridization. Pattern variations were calculated based on the model used for Fig. 3A, with different sets of parameter values. Spheres in the leftmost column and the top row represent “pure species” computed using uniform parameter values. Fused blobs at the crossing points represent “hybrids” resulting from in silico hybridization between parental spheres.

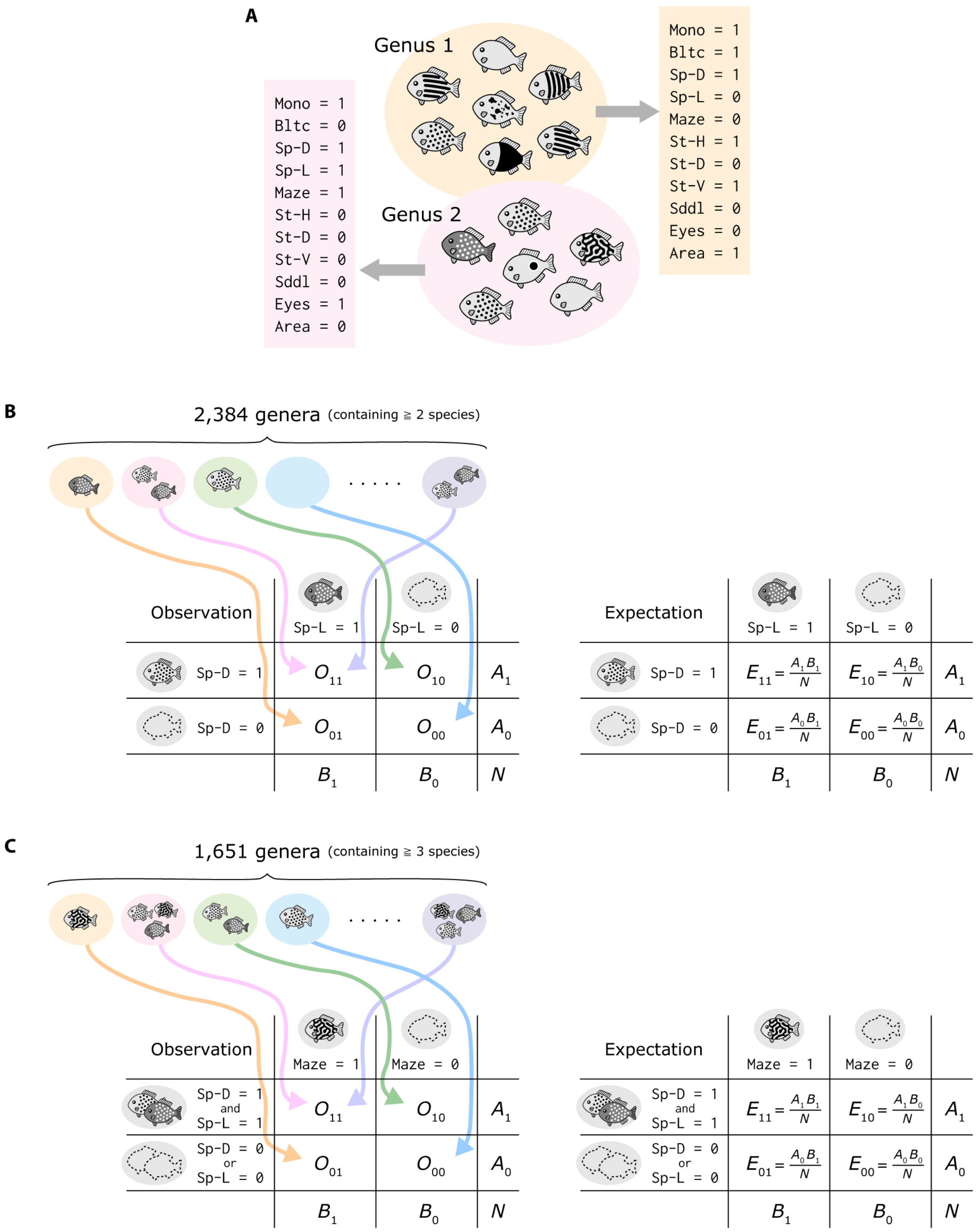

Fig. S2. Schematics of the binary encoding and co-occurrence analyses of pattern motifs. (A) Encoding of genus-wise pattern diversity. For each genus and each class of pattern motifs, the occurrence of species with that pattern motif was counted and binary labeled (1, containing species with the pattern motif; 0, not containing species with the pattern motif). **(B)** Co-occurrence analysis of pattern motifs. Here, a pair of motifs, Sp-D and Sp-L, is shown as an example. The occurrence or non-occurrence of each of these motifs was counted for N = 2,384 genera containing two or more species. O_{11} is the number of genera in which both of these motifs occur.

O_{10} and O_{01} are the numbers of genera in which only Sp-D or Sp-L occurs, respectively. O_{00} is the number of genera in which neither of them occurs. These are the observed frequencies. A_1 (B_1) and A_0 (B_0) are the numbers of genera in which Sp-D (Sp-L) occur and do not occur, respectively (regardless of the state of the other motif). E_{11}, E_{10}, E_{01}, and E_{00} are the expected frequencies calculated under the hypothesis that the occurrence of each motif is independent of each other. **(C)** Triple co-occurrence analysis of pattern motifs. Here, the case for a set of motifs (Sp-D, Sp-L, and Maze) is shown. A total of 1,651 genera containing three or more species were included in the calculation (see Methods for details).

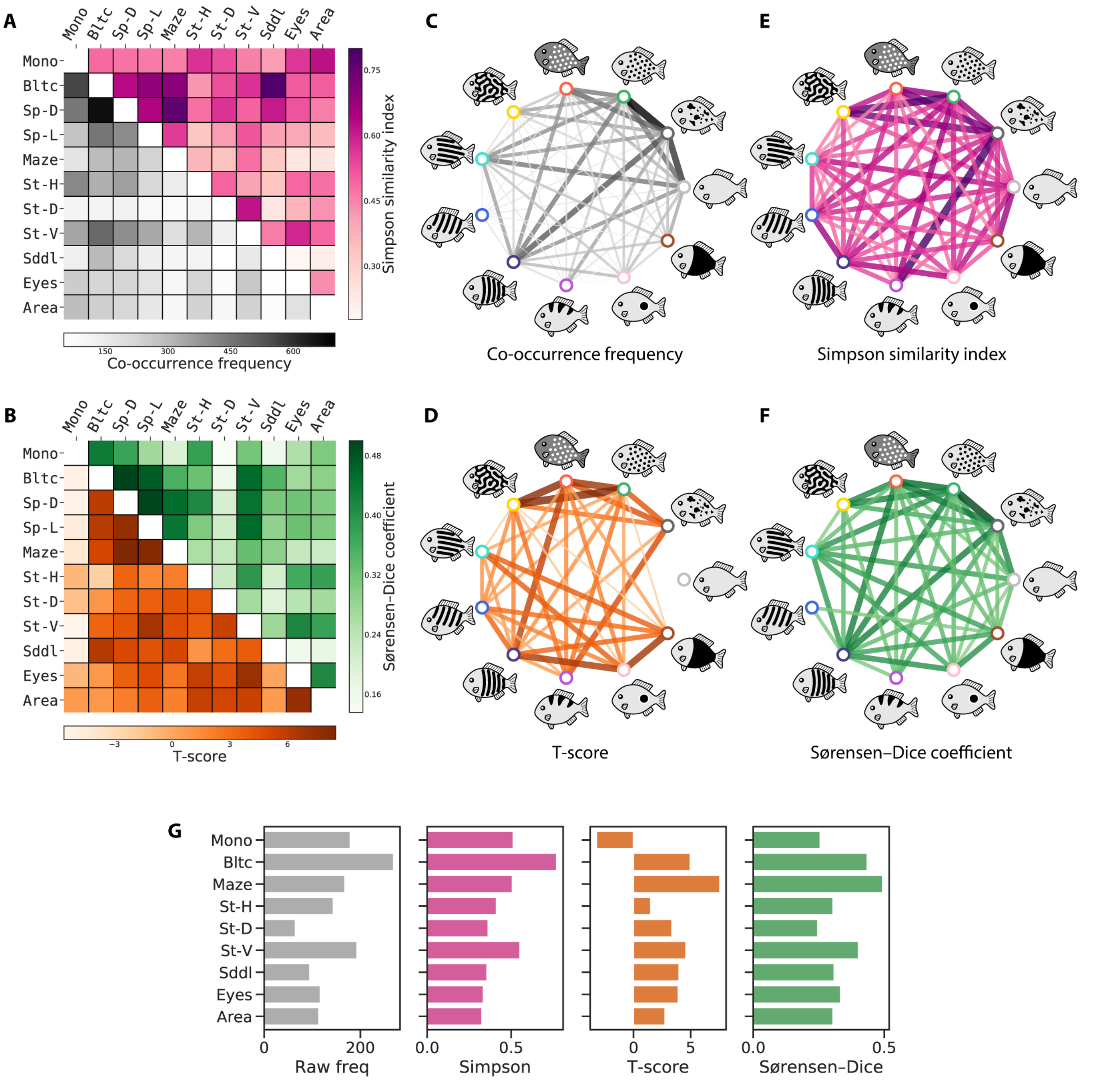

Fig. S3. Genus-level co-occurrences and mechanistic associations of color pattern motifs. (A and B) Heatmaps showing the frequency, strength and significance of the genus-level co-occurrence between each pairwise combination of the 11 pattern motifs evaluated using various association measures. **(C to F)** Mechanistic associations among pattern motifs summarized from the association matrices shown in (A and B). **(G)** Triple co-occurrence analysis showing the strength of the phylogenetic collocation of each pattern motif with a set of two spot motifs (Sp-D and Sp-L).

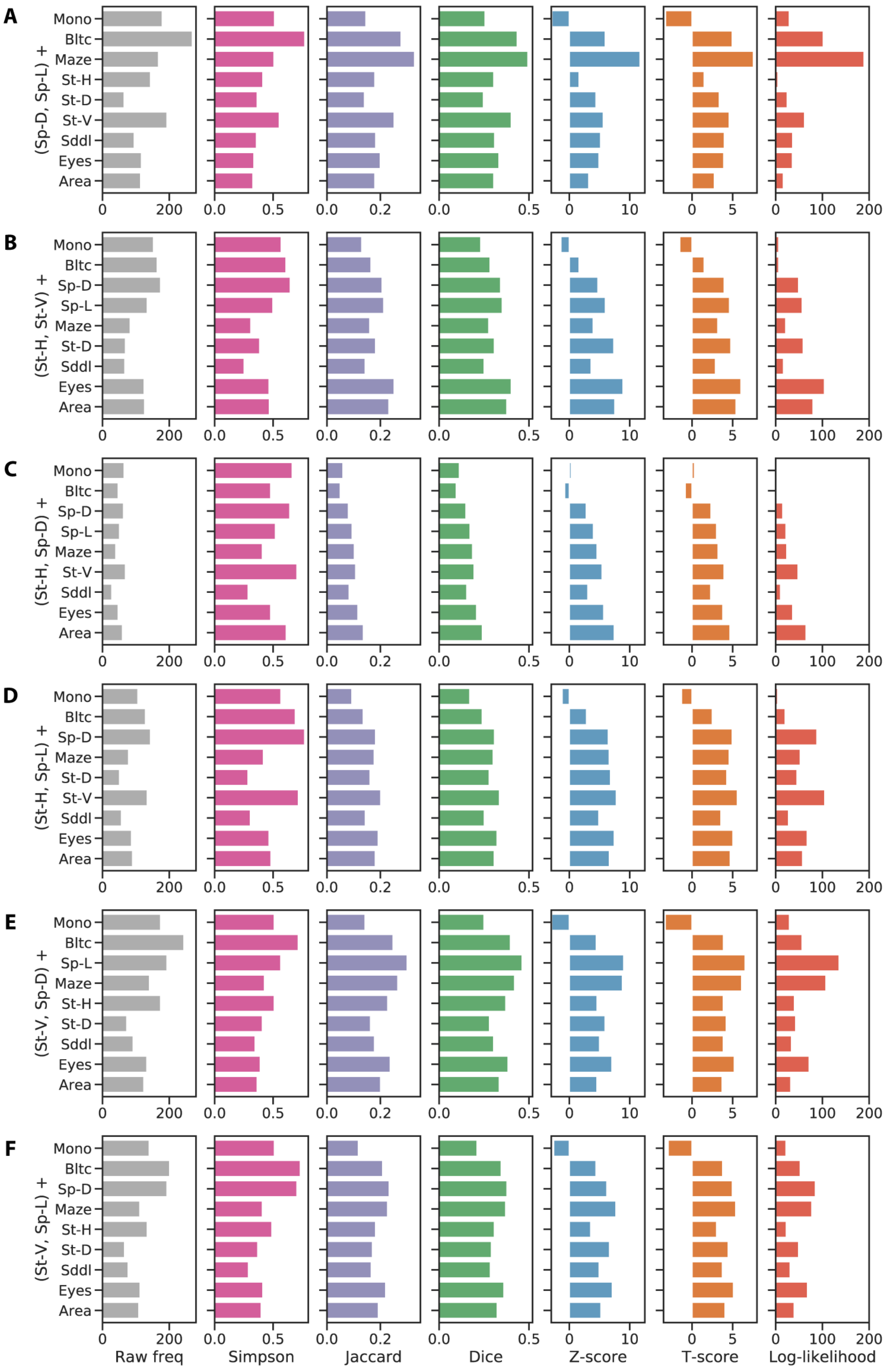

Fig. S4. Triple co-occurrence analyses for various combinations of motifs. The strength of the phylogenetic collocation between six sets of motif pairs (A to F) and each one of the other motifs was measured using six association measures.

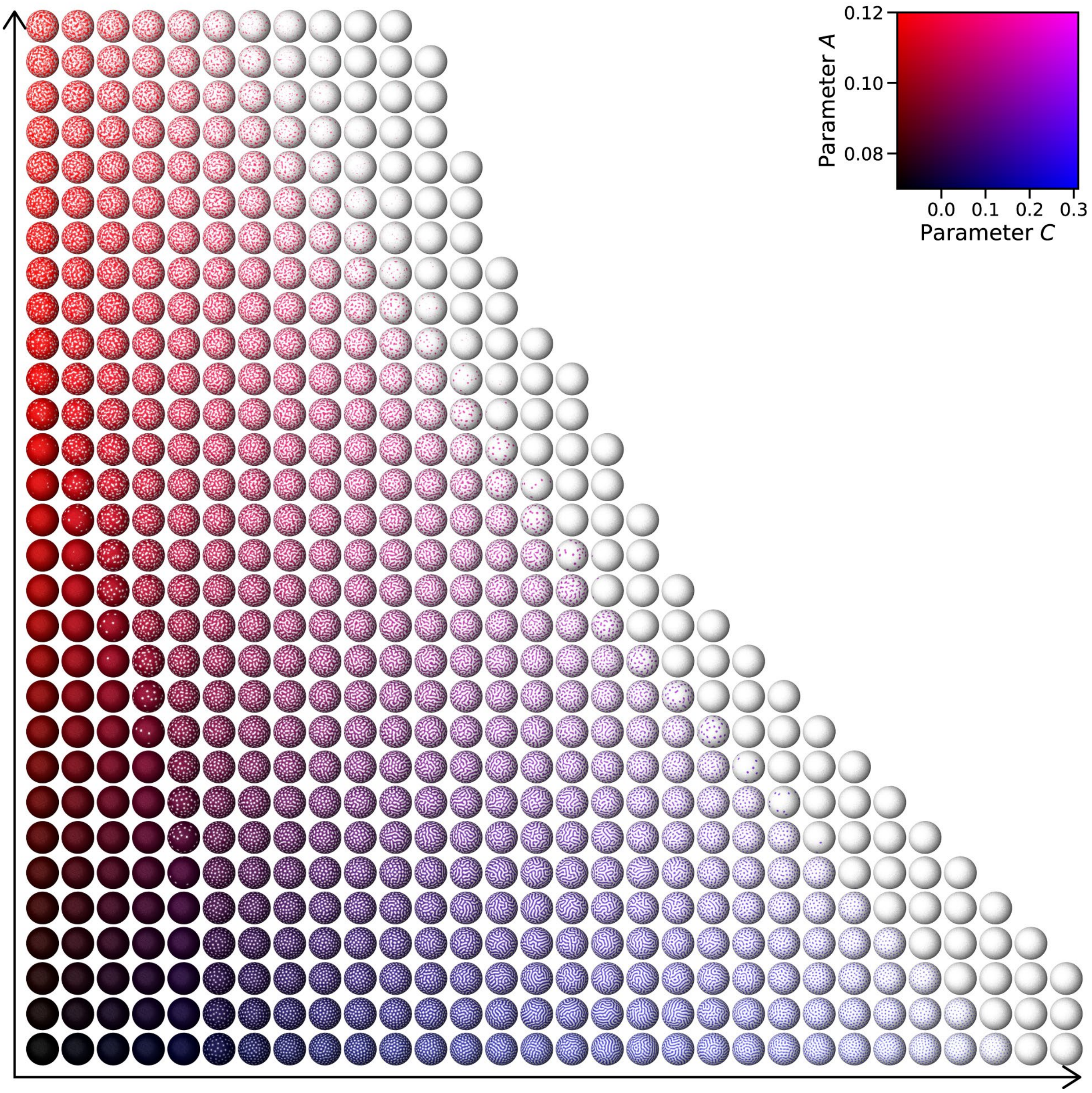

Fig. S5. Color patterns generated by the RD system. Pattern variations were calculated based on the model used for Fig. 3A, with different sets of uniform parameter values. Note that the color scheme is different from Fig. 3A for indicating multiple parameter values.

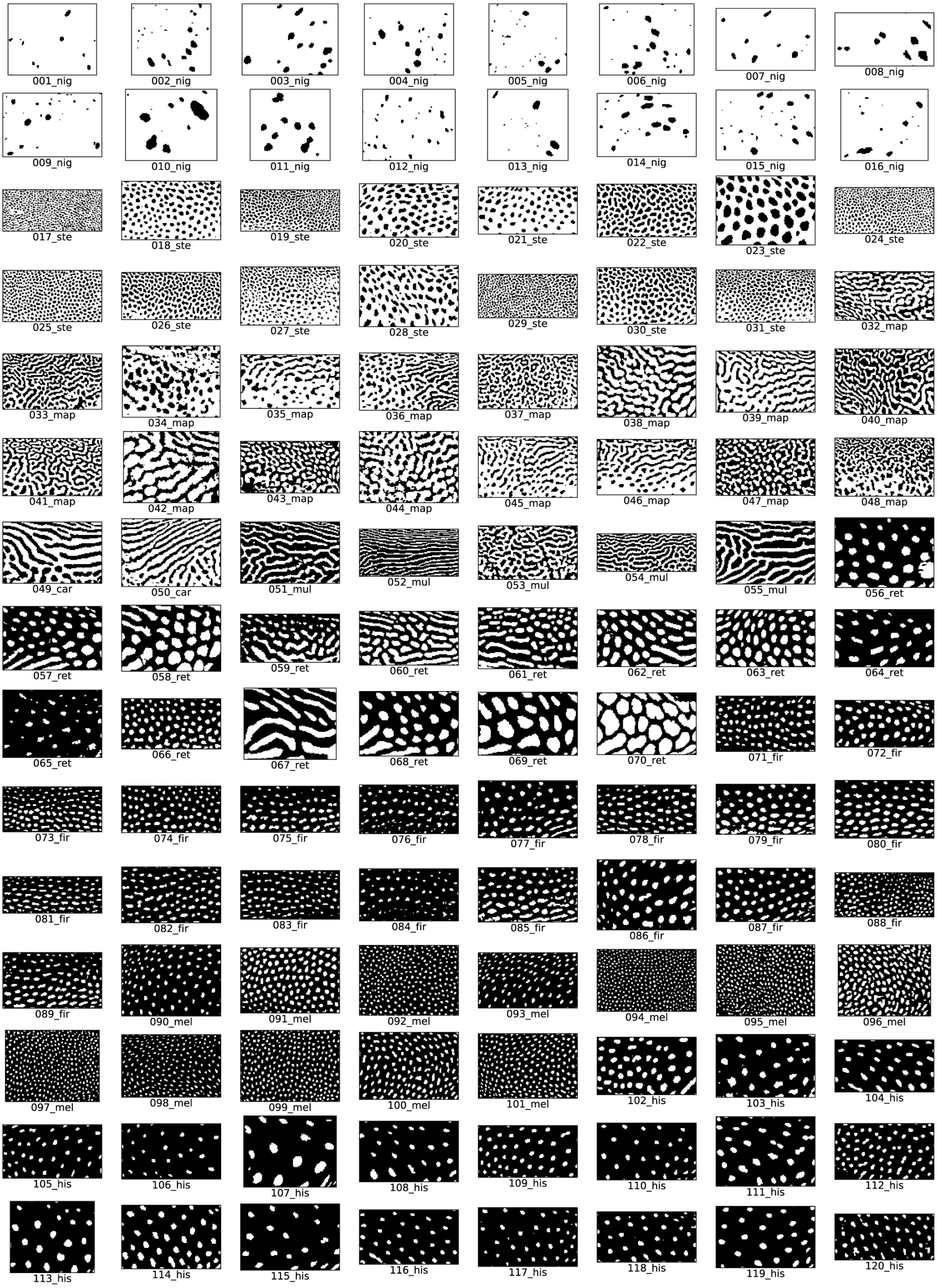

Fig. S6. Body patterns of *Arothron* species. Binarized images of body patterns of *Arothron* species used for pattern quantification analysis.

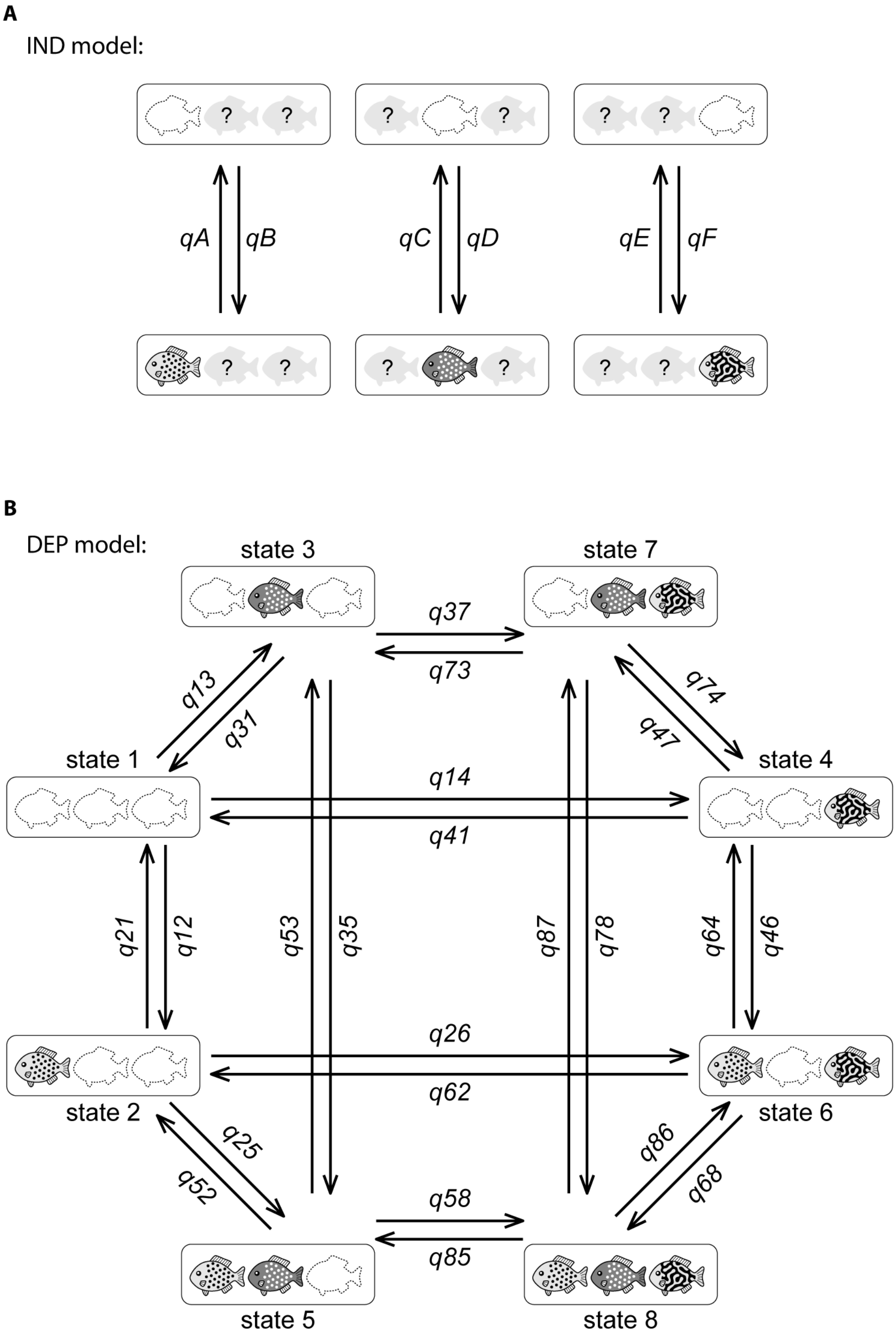

Fig. S7. Schematics of the independent and dependent models for pattern motif evolution. (A) The independent model: all the pattern motifs evolve independently without affecting one another. **(B)** The dependent model: the evolution of one motif can be affected by the state of other motifs. Twenty-four possible transitions are indicated by arrows. Double or triple transitions are not allowed in this model (e.g., transitions from state 1 to state 5, 6, 7 or 8 are set to zero).

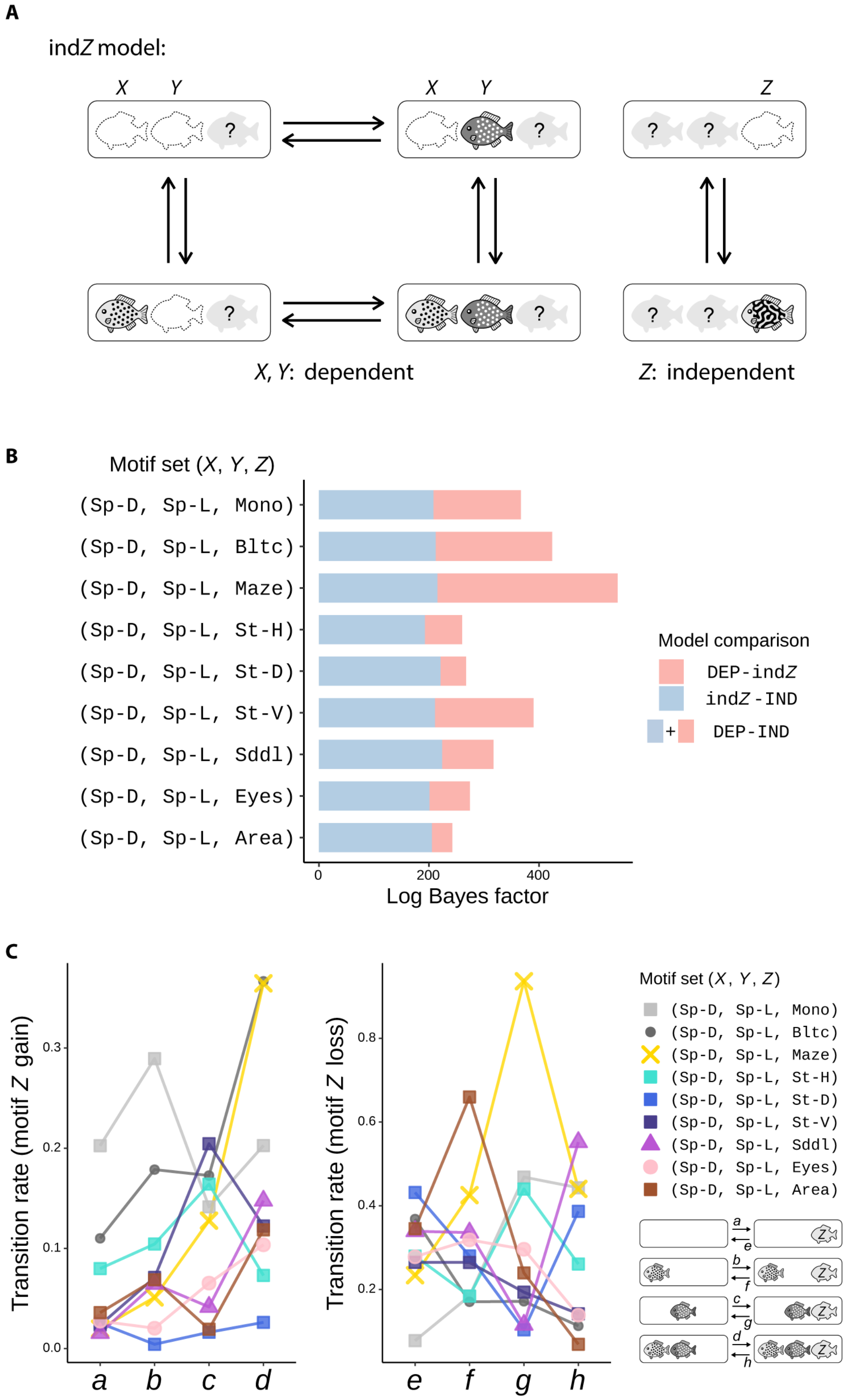

Fig. S8. Analysis of correlated evolution of dark and light spots and other motifs. (A) Schematic of the partially independent model: two motifs (*X* and *Y*) are correlated and evolve dependently, while the other motif (*Z*) evolves independently (ind*Z*). Here, the case for motif set (*X*, *Y*, *Z*) = (Sp-D, Sp-L, Maze) is shown. **(B)** Model comparison based on the Bayes factor. Blue bars indicate comparisons between the partially independent

model (ind*Z*) and the independent model (IND). This refers to the degree of support for correlated evolution between dark and light spots, which is about the same level for all motif combinations. Pink bars represent comparisons between the dependent model (DEP) and the partially independent model (ind*Z*). This refers to the degree of support for correlated evolution between motif *Z* and dark and light spots, which takes the highest value in the combination with the labyrinthine motif (Maze). **(C)** Estimated transition rates of motif *Z* under the dependent model. Bayesian rjMCMC analyses were performed on trait data available for the entire fish lineage (2,837 genera).

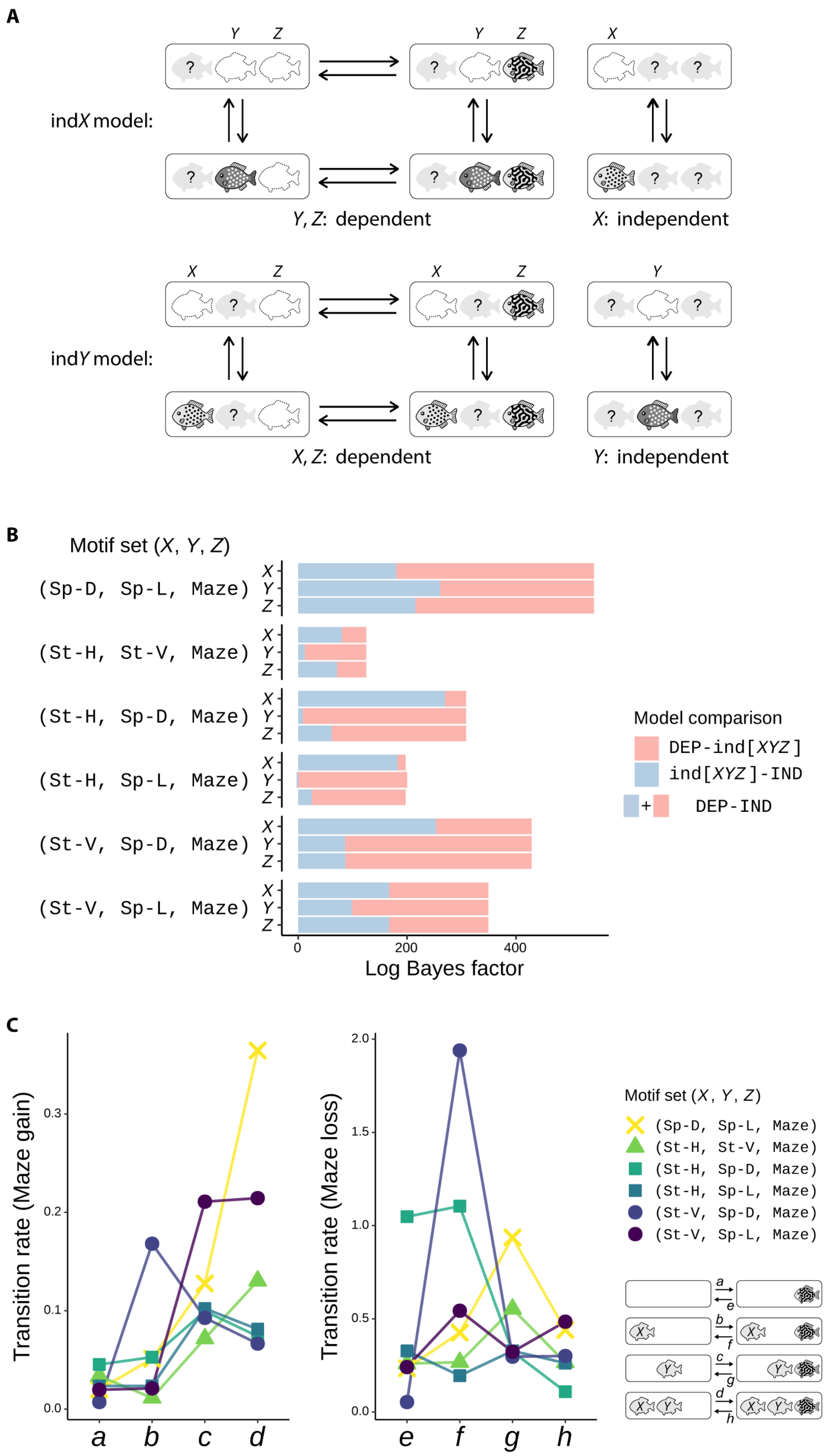

Fig. S9. Analysis of correlated evolution of the labyrinthine motif with other motif pairs. (A) Schematics of the partially independent models. Motif *X* evolves independently under the ind*X* model, while motif *Y* evolves

independently under the ind*Y* model. **(B)** Model comparison based on the Bayes factor. Blue bars show comparisons between the partially independent model (ind*X*, ind*Y*, or ind*Z*) and the independent model (IND), while pink bars show comparisons between the dependent model (DEP) and the partially independent model. A larger proportion of blue compared to pink indicates a relatively high degree of support for the partially independent model. For example, there is relatively high support for the ind*X* model in the motif set (St-H, Sp-D, Maze), suggesting correlated evolution between Sp-D and Maze and the independent evolution of St-H. **(C)** Estimated transition rates of the labyrinthine motif (Maze) under the dependent model. Bayesian rjMCMC analyses were performed on trait data available for the entire fish lineage (2,837 genera).

Table S1. *D* statistics (ABBA-BABA tests) and D_{min} statistics for trios of *Arothron* species. Patterson's *D* statistics were calculated using the *Dichotomyctere nigroviridis* genome (tetNig2) as an outgroup and reference. Significantly positive D_{min} scores are indicated in bold (Holm–Bonferroni FWER < 0.01).

A	*B*	*C*	*D(A,B;C,O) (Z)*	*D(A,C;B,O) (Z)*	*D(C,B;A,O) (Z)*	D_{min}
A. hispidus	*A. mappa*	*A. reticularis*	0.095 (39.39)	-0.377 (-155.59)	0.455 (193.86)	**0.095**
A. meleagris	*A. mappa*	*A. manilensis*	0.449 (70.54)	0.363 (55.40)	0.102 (33.23)	**0.102**
A. nigropunctatus	*A. mappa*	*A. manilensis*	0.452 (70.65)	0.367 (55.87)	0.102 (32.88)	**0.102**
A. diadematus	*A. mappa*	*A. manilensis*	0.449 (70.63)	0.365 (55.04)	0.101 (32.46)	**0.101**
A. immaculatus	*A. mappa*	*A. nigropunctatus*	0.094 (31.26)	-0.370 (-55.95)	0.449 (71.35)	**0.094**
A. immaculatus	*A. mappa*	*A. diadematus*	0.094 (31.07)	-0.369 (-55.72)	0.447 (70.84)	**0.094**
A. immaculatus	*A. mappa*	*A. meleagris*	0.094 (30.44)	-0.367 (-55.45)	0.445 (69.43)	**0.094**
A. firmamentum	*A. mappa*	*A. hispidus*	0.298 (63.40)	0.242 (47.95)	0.060 (28.17)	**0.06**
A. diadematus	*A. mappa*	*A. hispidus*	0.446 (86.10)	0.403 (73.71)	0.053 (24.38)	**0.053**
A. stellatus	*A. mappa*	*A. reticularis*	0.048 (23.91)	-0.431 (-196.29)	0.469 (210.02)	**0.048**
A. meleagris	*A. mappa*	*A. hispidus*	0.445 (85.15)	0.402 (74.43)	0.053 (23.82)	**0.053**
A. firmamentum	*A. mappa*	*A. reticularis*	0.163 (30.78)	-0.139 (-23.71)	0.295 (40.12)	**0.139**
A. nigropunctatus	*A. mappa*	*A. hispidus*	0.448 (86.90)	0.406 (74.42)	0.052 (23.45)	**0.052**
A. manilensis	*A. mappa*	*A. stellatus*	0.138 (30.98)	0.085 (21.28)	0.054 (26.41)	**0.054**
A. immaculatus	*A. mappa*	*A. stellatus*	0.135 (30.15)	0.083 (21.25)	0.052 (26.20)	**0.052**
A. firmamentum	*A. mappa*	*A. nigropunctatus*	0.290 (76.66)	-0.159 (-18.87)	0.429 (62.83)	**0.159**
A. firmamentum	*A. mappa*	*A. diadematus*	0.288 (76.60)	-0.157 (-18.47)	0.426 (61.74)	**0.157**
A. firmamentum	*A. mappa*	*A. meleagris*	0.288 (75.63)	-0.155 (-18.41)	0.424 (61.71)	**0.155**
A. firmamentum	*A. mappa*	*A. stellatus*	0.326 (61.80)	0.295 (59.14)	0.034 (17.13)	**0.034**
A. firmamentum	*A. mappa*	*A. manilensis*	0.153 (29.00)	0.230 (54.17)	-0.079 (-11.61)	**0.079**
A. firmamentum	*A. mappa*	*A. immaculatus*	0.155 (29.53)	0.231 (53.39)	-0.079 (-11.49)	**0.079**
A. diadematus	*A. mappa*	*A. meleagris*	-0.799 (-194.78)	0.020 (6.80)	-0.806 (-210.29)	**0.02**
A. nigropunctatus	*A. mappa*	*A. meleagris*	-0.816 (-214.19)	0.014 (6.59)	-0.820 (-216.40)	**0.014**
A. manilensis	*A. mappa*	*A. hispidus*	0.115 (29.14)	0.023 (6.21)	0.092 (38.28)	**0.023**
A. immaculatus	*A. mappa*	*A. hispidus*	0.114 (29.70)	0.023 (6.07)	0.091 (37.81)	**0.023**
A. hispidus	*A. mappa*	*A. stellatus*	0.009 (4.00)	0.069 (33.45)	-0.060 (-34.99)	**0.009**
A. nigropunctatus	*A. mappa*	*A. reticularis*	0.468 (73.90)	0.017 (3.65)	0.454 (125.52)	**0.017**
A. nigropunctatus	*A. mappa*	*A. diadematus*	-0.821 (-221.15)	-0.008 (-3.14)	-0.818 (-209.13)	**0.008**
A. immaculatus	*A. mappa*	*A. reticularis*	-0.430 (-76.96)	-0.419 (-114.13)	-0.013 (-2.82)	0.013
A. meleagris	*A. mappa*	*A. reticularis*	0.464 (73.34)	0.012 (2.57)	0.455 (128.05)	0.012
A. nigropunctatus	*A. mappa*	*A. stellatus*	0.456 (94.36)	0.460 (89.42)	-0.005 (-2.54)	0.005
A. diadematus	*A. mappa*	*A. reticularis*	0.465 (72.83)	0.012 (2.47)	0.456 (130.08)	0.012
A. manilensis	*A. mappa*	*A. reticularis*	-0.427 (-75.25)	-0.419 (-113.29)	-0.009 (-2.05)	0.009
A. diadematus	*A. mappa*	*A. stellatus*	0.454 (94.80)	0.457 (88.63)	-0.004 (-1.95)	0.004
A. meleagris	*A. mappa*	*A. stellatus*	0.454 (91.30)	0.456 (88.84)	-0.003 (-1.64)	0.003
A. immaculatus	*A. mappa*	*A. manilensis*	-0.784 (-173.93)	-0.003 (-1.44)	-0.783 (-172.12)	0.003

Table S2. Bayesian inference of correlated evolution of pattern motifs within major fish orders. Evidence for correlated evolution is indicated by asterisks [***: very strong evidence, log Bayes factor (BF) > 10; **: strong evidence, log BF > 5; *: positive evidence, log BF > 2]**.** N_{genera}: the number of genera investigated within each order; $N_{genMaze}$: the number of genera in which Maze motifs occur. Each MCMC analysis was run three times.

Order	N_{genera}	$N_{genMaze}$	Run	Log marginal likelihood Independent model	Log marginal likelihood Dependent model	Log Bayes factor
Cypriniformes	326	28	1	-309.55	-296.23	26.64 ***
			2	-309.33	-297.64	23.39 ***
			3	-309.70	-297.13	25.13 ***
Siluriformes	301	33	1	-380.04	-368.80	22.50 ***
			2	-380.75	-369.56	22.36 ***
			3	-379.28	-371.40	15.75 ***
Perciformes	260	51	1	-457.56	-434.34	46.44 ***
			2	-458.33	-435.89	44.87 ***
			3	-456.99	-437.93	38.11 ***
Cichliformes	181	19	1	-270.31	-263.65	13.33 ***
			2	-270.77	-264.80	11.94 ***
			3	-270.21	-264.82	10.78 ***
Gobiiformes	153	29	1	-289.57	-277.50	24.14 ***
			2	-289.88	-277.90	23.96 ***
			3	-289.78	-277.82	23.92 ***
Characiformes	143	2	1	-100.95	-100.91	0.09
			2	-102.06	-101.30	1.52
			3	-101.08	-101.68	-1.19
Cyprinodontiformes	99	18	1	-177.86	-170.23	15.26 ***
			2	-177.67	-169.72	15.90 ***
			3	-177.30	-169.97	14.66 ***
Blenniiformes	84	22	1	-152.03	-152.01	0.05
			2	-151.58	-152.10	-1.05
			3	-151.91	-151.92	-0.02
Pleuronectiformes	82	9	1	-146.70	-138.33	16.73 ***
			2	-146.54	-137.80	17.48 ***
			3	-146.72	-136.94	19.57 ***
Tetraodontiformes	70	35	1	-142.30	-140.30	4.00 *
			2	-142.30	-140.33	3.94 *
			3	-142.31	-140.01	4.60 *

Data file S1 (separate file). Color pattern annotation dataset and raw results of pattern quantification. (A) Color pattern annotation dataset for the images of 18,114 fish species. **(B)** The presence/absence of each pattern motif compiled for each species. **(C)** The presence/absence of each pattern motif compiled for each genus. **(D)** The presence/absence of each pattern motif compiled for each family. **(E)** Raw results of pattern quantification analysis with links/credits for the original images.
